# Development of a Cancer-Associated Fibroblast-Related Prognostic Model in Breast Cancer via Bulk and Single-Cell RNA Sequencing

**DOI:** 10.1155/2022/2955359

**Published:** 2022-12-02

**Authors:** Jing Hu, Yueqiang Jiang, Qihao Wei, Bin Li, Sha Xu, Guang Wei, Pin Li, Wei Chen, Wenzhi Lv, Xianjin Xiao, Yaping Lu, Xuan Huang

**Affiliations:** ^1^Huangshi Hubei Medical Group of Maternal and Child Health Hospital, Hubei, China; ^2^Department of Geriatrics, Tongji Hospital, Tongji Medical College Huazhong University of Science and Technology, Wuhan, China; ^3^Sinopharm Genomics Technology Co., Ltd, Wuhan, China; ^4^Department of Artificial Intelligence, Julei Technology Company, Wuhan, China; ^5^Institute of Reproductive Health, Tongji Medical College, Huazhong University of Science and Technology, Wuhan, China; ^6^Department of Ophthalmology, Beijing Chao-Yang Hospital, Capital Medical University, Beijing, China; ^7^Medical Research Center, Beijing Chao-Yang Hospital, Capital Medical University, Beijing, China

## Abstract

**Background:**

The most numerous cells in the tumor microenvironment, cancer-associated fibroblasts (CAFs) play a crucial role in cancer development. Our objective was to develop a cancer-associated fibroblast breast cancer predictive model.

**Methods:**

We acquire breast cancer (BC) scRNA-seq data from Gene Expression Omnibus (GEO), and “Seurat” was used for data processing, including quality control, filtering, principal component analysis, and t-SNE. Afterward, “singleR” software was used to annotate cells. Seurat's “FindAllMarkers” program is used to locate particular CAF markers. clusterProfiler was used to analyze Gene Ontology (GO) and Kyoto Encyclopedia of Genes and Genomes (KEGG) pathway enrichment. The Cancer Genome Atlas (TCGA) database was utilized to provide univariate Cox regression, least absolute shrinkage operator (LASSO) analysis using bulk RNA-seq data. For model development, multivariate Cox regression studies are used. Utilizing pRRophetic and Tumor Immune Dysfunction and Exclusion (TIDE) algorithms, chemosensitivity and immunotherapy response were predicted. The “rms” software was used to facilitate and simplify modeling.

**Results:**

Integrating the scRNA-seq (GSE176078) dataset yielded 28 cell clusters. In addition, well-known cell types helped identify 12 cell types. We found 193 marker genes that are elevated in CAFs. In addition, a five-gene predictive model associated to CAF was created in the training set. In the training set, the validation set, and the external validation set, greater risk scores were associated with a worse prognosis. And individuals with a higher risk score were more susceptible to immunotherapy and conventional chemotherapy medicines.

**Conclusion:**

In conclusion, we establish a strong prognostic model comprised of 5 genes related with CAF that might serve as a potent prognostic indicator and aid clinicians in making more rational medication choices.

## 1. Introduction

Cancer continues to be a significant worldwide health concern and the main cause of death in China [1, 2]. According to the 2015 China Cancer Epidemiology Report [3], breast cancer was the most commonly diagnosed cancer among Chinese women in 2015, with an estimated 304,000 new cases, or more than 800 new cases each day. Additionally, the incidence of breast cancer is rising by around 0.5% every year [4]. Delayed diagnosis may result in an advanced disease stage upon presentation [5]. With the advent of surgical surgery and associated adjuvant therapy, early diagnosis has significantly improved patient outcomes.

Immune responses in the microenvironment of the tumor are also thought to have a significant role in determining the aggressiveness and development of the tumor. As a result of tumor heterogeneity and complicated tumorigenic pathways, it is very difficult to establish tailored treatment plans and reliably anticipate patient outcomes [6, 7]. The propensity of CAFs to promote tumor growth makes them a potential immunotherapy target, according to studies [8–10]. The mechanism of CAF in tumors has been intensively explored, although its relevance to the prognosis of tumors is still unknown.

In this research, we evaluated single cell RNA-seq data from a breast cancer patient and discovered 193 fibroblast markers with highly variable expressions. Using the TCGA-BRCA cohort data set, we created a unique CAF-related signature model with excellent robustness that can correctly discriminate between patients with high and low risk. Then, we confirmed that the five-gene model could accurately predict prognosis and therapeutic response. Analyses of univariate and multivariate Cox regression confirmed the CAF-related signature (or risk score) as an independent risk factor for OS. In order to improve the prediction effectiveness of the signature and enable clinical application, we subsequently created and validated a nomogram based on age, TNM stage, and CAF signature for clinical applicability in order to predict OS. The quantity of CD8^+^T cells in malignancies influences the efficacy of the majority of immunotherapies [11, 12]. And according to our results, the CD8^+^T cell infiltration rate was greater in patients with a high-risk score. We discovered that patients with a high-risk score were more likely to react to anti-PD-1 and anti-CTLA-4 therapy than those with a low-risk score. In addition, the pRRophetic algorithm revealed that patients with a high-risk score were more responsive to various conventional chemotherapy drugs than those with a low-risk score.

## 2. Materials and Methods

### 2.1. The Data Source

The scRNA-seq data of 26 breast cancer patient tissues were obtained from GSE176078 through the Gene Expression Omnibus (GEO) database (http://www.ncbi.nlm.nih.gov/geo/) [13]. The samples were mostly from three clinical subgroups of breast cancer (11 ER+, 5 HER2, and 10 TNBC). The 10X Genomics platform was used to do single-cell sequencing. The bulk RNA-seq data and clinical information for the samples in The Cancer Genome Atlas Breast Invasive Carcinoma (TCGA-BRCA) cohort were retrieved via the UCSC Xena browser (https://xena.net/) [14], and 835 samples having survival information were recruited. The type of bulk RNA-seq data we use is FPKM. The ratio of training set to validation set is arbitrarily determined to be 7 : 3. The GEO database was queried for externally verified bulk RNA-seq breast cancer data (GSE20685). All analyses in this article were conducted using R version 4.1.2.

### 2.2. Analysis of a Single Cell Using RNA Sequencing

R software application Seurat [15] was used to analyze scRNA-seq data. In the first step of data quality check, cells with “nFeature” less than 200 and “percent.mt” less than 20% were filtered out. Then, single-cell data from several samples were merged and the batch effect was removed from the data. The “LogNormalize” approach was used to normalize the data before to unsupervised clustering of cells by principal component analysis (PCA), dimensionality reduction, and visualization by t-Distributed Stochastic Neighbor Embedding (t-SNE). The SingleR software package [16] was used to annotate each cell cluster's cell type. The “FindAllMarkers” program was used to discover differentially expressed marker genes among various cell types. The log2 fold change (FC) threshold value was less than 0.25, and “min.pct” equaled 0.25.

### 2.3. Analysis of Gene Function Enrichment

The “clusterProfiler” R package (V3.14.3) conducted Gene Ontology (GO) [17] and Kyoto Encyclopedia of Genes and Genomes (KEGG) [18] pathway enrichment analyses [19]. To identify marker genes in the cell cluster of interest for biological process (BP), molecular function (MF) and cellular component (CC) enrichment at *p* < 0.05 significance level.

### 2.4. Development of a CAF-Related Prognostic Model

This study's main endpoint was overall survival (OS), and univariate Cox regression analysis was performed to filter potential genes related with prognosis from cancer-associated fibroblasts (CAFs) genes in the training set using a *p* < 0.05 threshold. In order to decrease the possibility of overfitting, we subsequently evaluated prognostic candidate genes using the least absolute shrinkage operator (LASSO) Cox regression model in the “glmnet” R package [20]. Then, a stepwise backward selection strategy based on the Akaike information criterion (AIC) was employed to get significant variables [21] in order to exclude unsuitable prognostic models for CAF. The CAF-related risk score was computed as follows:

(1) $$\begin{array}{c} \text{risk score}=\sum \left(\beta_{i}\times {\text{Exp}}_{i}\right) \end{array}$$

where *β*_*i*_ denotes the coefficient of LASSO regression for the genes, and Exp_*i*_  denotes the expression value of the candidate gene. The “maxstat” R package approach was used to establish the appropriate cutoff for the grouping of risk score, and the patients were categorized into high-risk and low-risk groups, respectively.

### 2.5. Prediction of Chemotherapy Responsiveness and Immunotherapy Efficacy

To estimate the sensitivity of chemotherapeutic medicines in high-risk and low-risk groups, we extrapolated the half-maximal inhibitory concentration (IC50) of chemotherapeutic agents using the “pRRophetic” R package [22]. The experimental information for chemotherapeutic medicines (docetaxel, gemcitabine, paclitaxel, camptothecin, pazopanib, and sunitinib) was collected from the Genomics of Drug Sensitivity in Cancer (GDSC) database (https://www.cancerrxgene.org). In addition, the Tumor Immune Dysfunction and Exclusion (TIDE) (http://tide.dfci.harvard.edu/) algorithm [23] is employed to forecast the treatment response of two groups of Immune check point blocking.

### 2.6. Construction and Validation of Nomograms

Clinicopathological factors related with prognosis were identified using univariate Cox regression analysis, with the derived hazard ratio (HR). The variables with *p* values 0.05 were checked, and the prognosis risk score was calculated using the “rms” R software tool. The calibration curves were used to characterize the congruence between the actual data and the projected OS probability.

### 2.7. Statistical Analysis

This research used R software version 4.1.2 (https://www.r-project.org/) for statistical analysis and data visualization. The Wilcoxon test was used to compare the two groups. Using a two-sided log-rank test, the statistical significance of the difference in the overall survival (OS) of patients between the high-risk and low-risk groups was determined. For survival analysis, the packages “survival” [24] and “survminer” were used. A *p* value 0.05 was regarded as statistically significant.

## 3. Results

### 3.1. Identification of Fibroblasts Pertinent to Cancer

**Figure 1:**
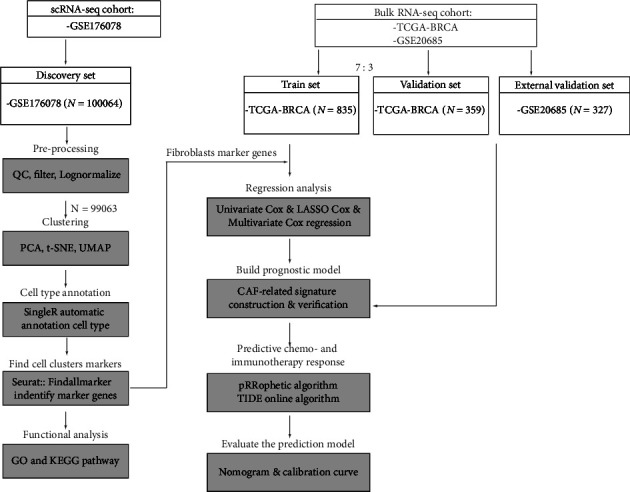
The study's schematic diagram.

**Figure 2:**
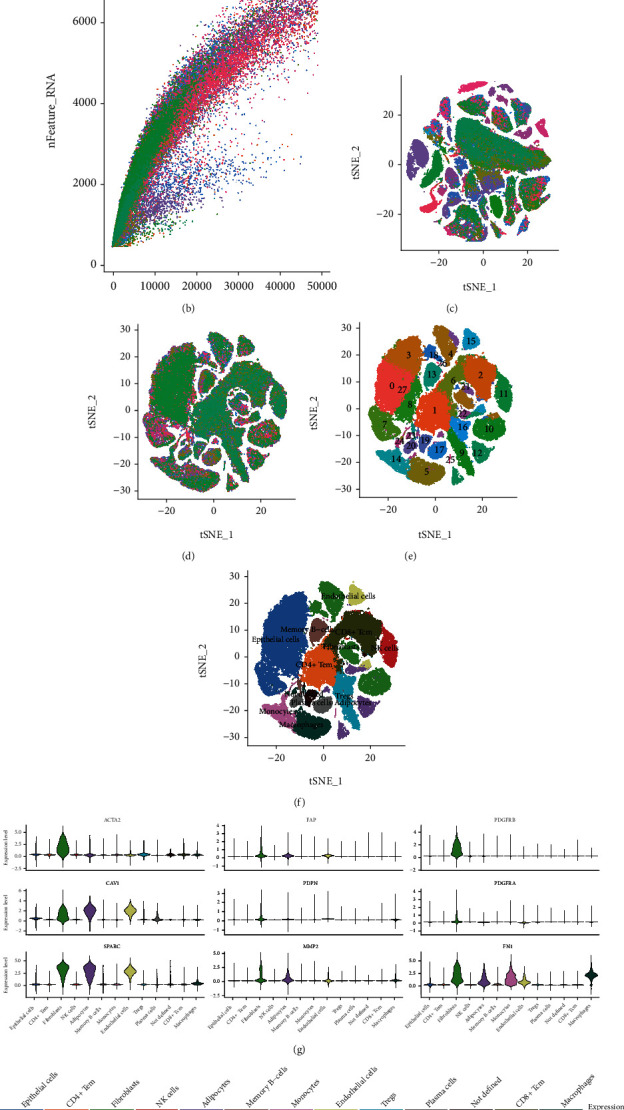
Analysis of single-cell RNA sequencing. (a) RNA characteristic number (nFeature RNA) and absolute UMI count (nCount RNA) were presented using violin diagram after quality control screening of cells. (b) Analysis of the correlation between nFeature and nCount. (c) Before eliminating batch effect, visual T-SNE clustering was separated into 26 samples, with each color representing one sample. (d) After eliminating the batch effect, T-SNE clustering was displayed using 26 samples, with each hue representing a single sample. (e) After eliminating the batch effect, T-SNE clusters were organized into clusters of cells, with each hue representing a cluster of cells. (f) After batch effect is eliminated, visual T-SNE clusters are sorted by known annotated cell types, with each color representing a cell type. (g) Violin graphic depicts the expression of nine identified fibroblast marker genes in each cell type. (h) Heat map depicting the expression of the top ten marker genes in each of the twelve identified cell types.

Figure 1 depicts our study process in its entirety. The scRNA-seq (GSE176078) data of 26 breast cancer tissue samples were downloaded from the GEO database. Low-quality cells (thresholds “nFeature RNA” >200 and “percent.mt” 20%) were filtered out (Figure 2(a)) and 99,063 high-quality cells were identified. The expression matrix was then subjected to standardization. There was a significant positive association between the number of discovered genes (nFeature) and the sequencing depth (number of UMIs, nCount) (Figure 2(b)). In the meanwhile, the “LogNormalize” technique was used to standardize the data. ANOVA was used to identify highly variable genes, and the top 2,000 highly variable genes were chosen for further investigation (Supplementary Figure S1).

The batch effect is often present in scRNA-seq data of greater magnitude, which may impact data integration and interpretation. As seen in Figure 2(c), samples exhibit batch effect. Several groups of cells from a single sample suggest that the significant discrepancies between these clusters may be attributable to sequencing batch. Given this, we merged the 26 samples and eliminated the batch effect. Subsequently, t-SNE clustering analysis was done on the first 20 main components. Finally, 99, 063 cells were grouped into 28 cell clusters from 26 samples (Figure 2(e)). After eliminating the batch effect, the visual clustering results of grouping data by source revealed that the difference in sample source was no longer the primary distinction between all cell groups (Figure 2(d)).

To determine the cell type of each cluster, the cell cluster was annotated with SingleR. Epithelial cells and CD4+ Tem, fibroblasts, NK cells, adipocytes, memory B cells, monocytes, endothelial cells, Tregs, plasma cells, CD8 cells+ Tcm, macrophages, and not defined cell constituted the majority of the described cell types (Figure 2(f)). We employed established fibroblast marker genes (*ACTA2, FAP, PDGFRB, CAV1, PDPN, PDGFRA, SPARC, MMP2,* and *FN1)* to validate the annotations [25, 26]. As demonstrated in Figure 2(g), fibroblasts expressed marker genes at high levels. In addition, “FindAllMarkers” was utilized to discover marker genes with differential expression among cell clusters. A random sample of one thousand cells was taken from each cell cluster to depict the top 10 differentially expressed genes using heat maps (Figure 2(h)). In accordance with the criteria (logFC > 0.25 and adj *p* value < 0.05), we identified a total of 193 marker genes that were differently expressed in fibroblast cluster relative to other cell clusters. Consequently, we displayed the gene expression of nine markers using violin plot (Supplementary Figure S2).

### 3.2. GO and KEGG Enrichment Analysis

**Figure 3:**
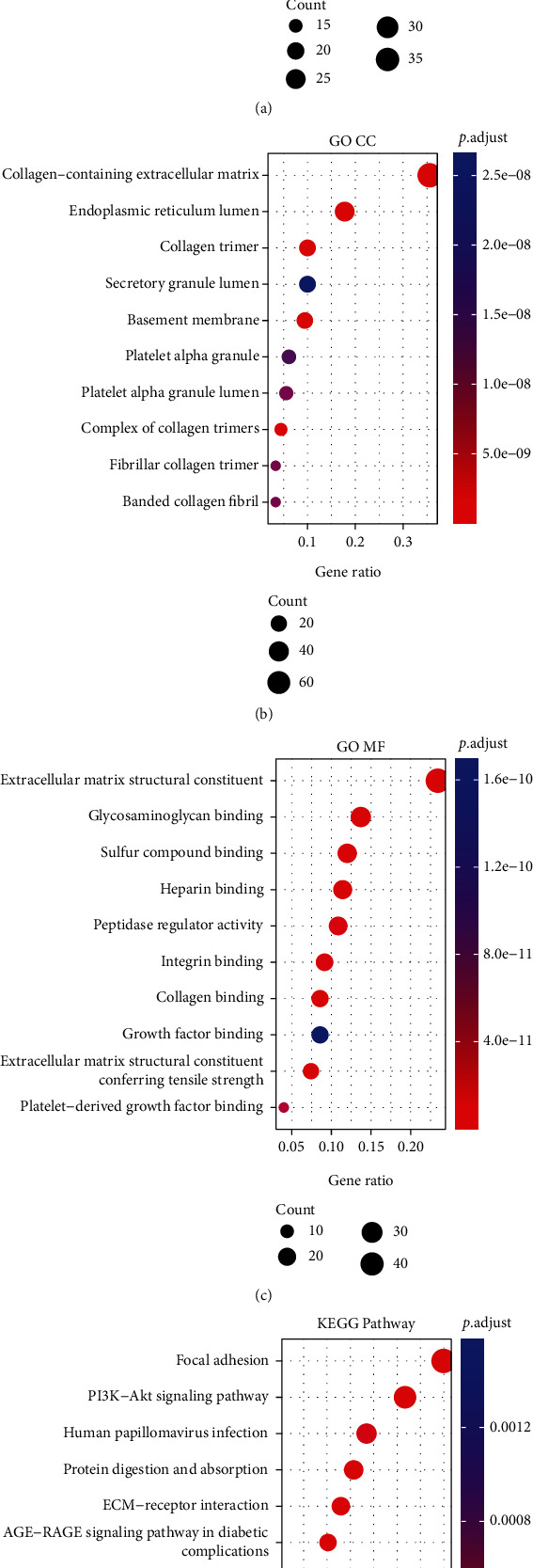
Analysis of enrichment of GO and KEGG pathways. (a) The bubble diagram of GO enrichment analysis displays the top 10 biological processes (BP) that are enriched. (b) The top 10 cells enriched in cellular component (CC) are shown in the bubble diagram of GO enrichment analysis. (c) The GO enrichment analysis bubble graphic depicts the ten most enriched molecular functions. (d) KEGG enrichment study of the top 10 pathways.

Using “clusterProfiler,” Gene Ontology (GO) enrichment analysis and Kyoto Encyclopedia of Genes and Genomes (KEGG) pathway analysis were performed to determine the unique biological relevance and critical pathways associated with 193 marker genes linked with cancer fibroblasts. As shown in Figures 3(a)–3(c), CAF marker genes were mostly enriched in extracellular matrix organization, extracellular structure organization, control of peptidase activity, wound healing, collagen fibril organization, and collagen metabolic process.  Figure 3(d) primarily depicts the top 10 pathways identified by KEGG enrichment analysis. Focal adhesion, PI3KAkt signaling pathway, human papillomavirus infection, protein digestion and absorption, ECM receptor interaction, proteoglycans in cancer, complement and coagulation cascades, relaxin signaling pathway, and amoebiasis. These enrichment findings revealed that the marker genes we screened were associated with fibroblast function, demonstrating that the genes we tested were credible fibroblast marker genes. Separate information on GO and KEGG enrichment findings is provided in Supplemental Table S1 and S2.

### 3.3. Prognostic Model Construction

**Figure 4:**
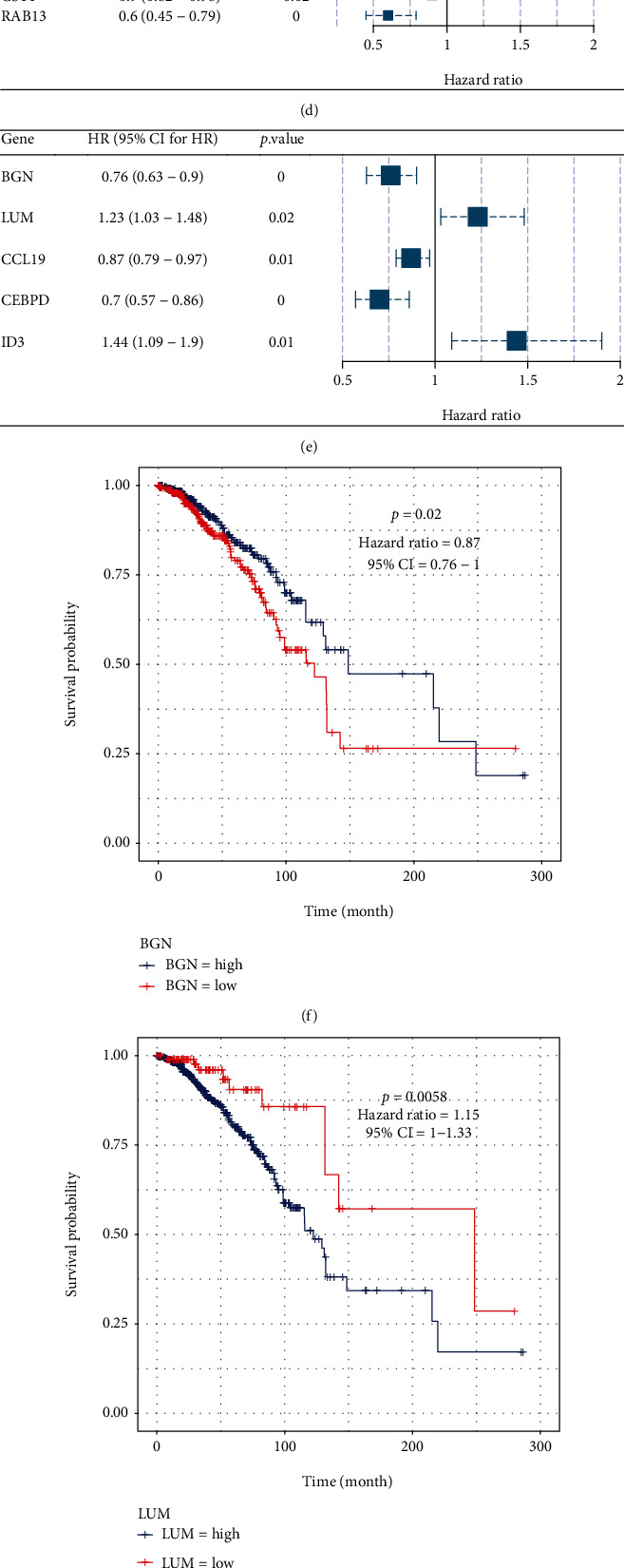
Prognostic model construction. (a) 10-fold cross-validation to determine the optimal lambda parameters. (b) LASSO model coefficient derived by optimum lambda. (c) Coefficients of the univariate Cox regression model for 15 prognostic genes. (d) Forest map depicting the hazard ratio (HR) and *p* value derived from a univariate Cox regression analysis. (e) Forest plot displaying hazard ratio (HR) and *p* value using multivariate Cox regression analysis. (f–j) Display of Kaplan-Meier survival curves of patients separated into high expression group and low expression group based on the expressions of *BGN*, *LUM*, *CEBPD*, *ID3*, and *CCL19*, respectively.

The TCGA-BRCA cohort (*N* = 835)was randomly split into training set and validation set in a 7 : 3 ratio. The 193 CAF genes in the training set were examined using univariate Cox regression (Supplementary Table S3), which identified 43 candidate genes as prognosis-related. These candidate genes were subsequently evaluated using LASSO Cox regression analysis with 10-fold cross-validation, and “lambda.min” was determined to be the best lambda value (Figures 4(a) and 4(b)). The model included 15 coefficients that were not zero (Figure 4(c)), indicating that 15 out of 43 factors may better predict clinical outcomes. Several of these genes, including *DSTN, ID*3*, TFPI, C*1*QTNF*1*, CCL*2*, EFEMP*1*, LUM,* and *FILIP*1*L*, have been identified as oncogenes with a hazard ratio (HR) > 1. In addition, *CXCL*9, *CST*1, *TIMP*1, *BGN*, *RAB*13, *CCL*19, and *CEBPD* were considered protective genes with HR < 1. (Figure 4(d)). To develop a model that can predict the prognosis of patients, we incorporated all 15 prognostic genes identified by LASSO regression into a multivariate Cox regression analysis model and used a stepwise backward algorithm to select the optimal model based on the Akaike information criterion (AIC). The best model was comprised of 5 genes; *BGN*, *LUM*, and *CEBPD* were protective genes (HR < 1, *p* < 0.05) (Figures 4(f)–4(h)), whereas *CCL19* and ID3 were risk genes (HR > 1, *p* < 0.05) (Figures 4(i), 4(j), and 4(e)). Based on these 5 genes, a prognostic model was developed: the integrated risk score = (0.38^∗^ exp (*BGN*)) + (0.27^∗^ exp (*LUM*)) + (0.16^∗^ exp (*CCL*19)) + (0.3^∗^ exp (*CEBPD*)) + (0.51^∗^ exp (*ID*3)).

### 3.4. Validation of Prognostic Model Performance

**Figure 5:**
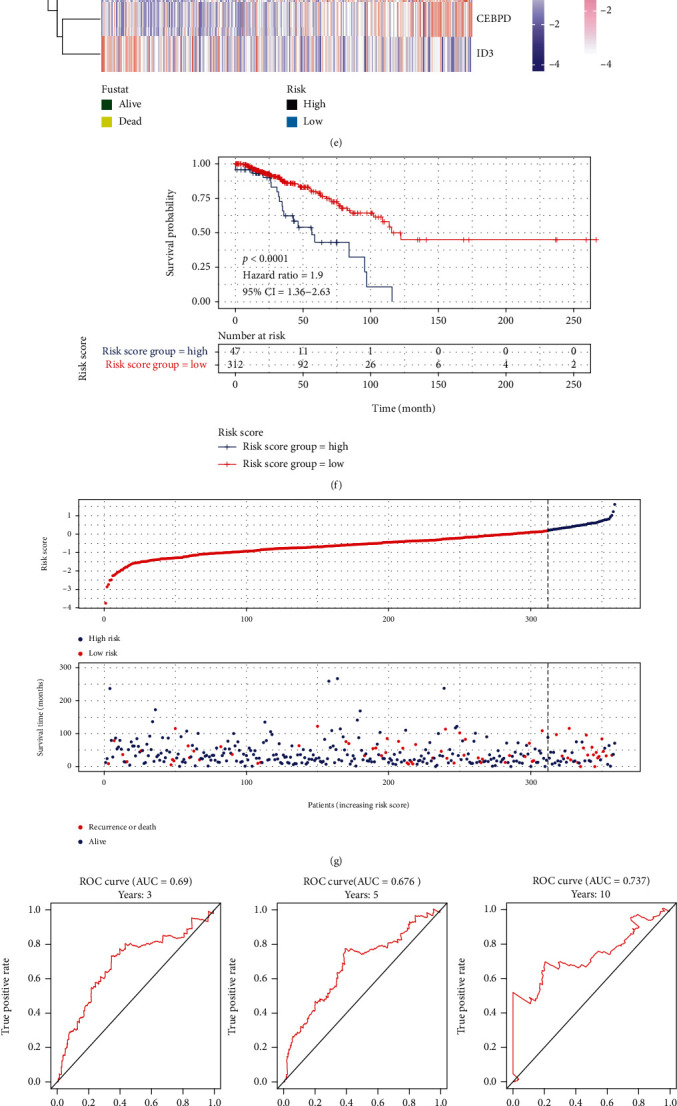
Validation of prognostic model performance. (a, e, and i) The heat map depicts the expressions of *BGN*, *LUM*, *CEBPD*, *CCL19*, and *ID3* in the training set, validation set, and external test set as well as the risk score grouping information. (b, g, and k) The scatter figure illustrates the distribution of sample risk scores and the survival status of patients in the training set, test set, and external test set, respectively. (c, f, j) The Kaplan-Meier graphs depict the survival of patients in the high-risk and low-risk categories of the training set, test machine, and external test set, respectively. (d, h, l) The ROC curves for predicting 3, 5, and 10-year survival from the training set, the test set, and the external test set, respectively, are shown by the curves.

Using the prognostic model, we estimated the risk score for the training set, validation set, and external validation set, and then separated the patients into high- and low-risk groups based on the risk score and the optimal cutoff value. A heat map depicted the risk score distribution, and the expression levels of five genes were included into the model for various data sets (Figures 5(a), 5(e), and 5(i)). Moreover, scatter plots illustrated the risk score and associated survival status of patients in each data set (Figures 5(b), 5(g), and 5(k)). To determine if risk score was connected with patient prognosis, we compared K-M survival curves across groups with high- and low-risk scores using the log-rank test. Patients in the high-risk category were shown to have a poor prognosis (train set: log-rank test *p* < 0.0001, HR = 2.72; validation set: log-rank test *p* < 0.0001, HR = 1.9; external validation set: log-rank test *p* < 0.0001, HR = 2.58). The area under the ROC curve (AUC) in the training set was 0.676 (3 years), 0.687 (5 years), and 0.749 (10 years) when the model was used to predict the 3-year, 5-year, and 10-year survival, respectively (Figure 5(d)). The AUC of the validation set was 0.69 (3 years), 0.676 (5 years), and 0.737 (10 years) (Figure 5(h)), while the AUC of the external validation set was 0.67 (3 years), 0.62 (5 years), and 0.621 (10 years) (Figure 5(l)). These findings suggested that the risk score is a reliable predictor.

### 3.5. Risk Score May Predict Response to Chemotherapy and Immunotherapy

**Figure 6:**
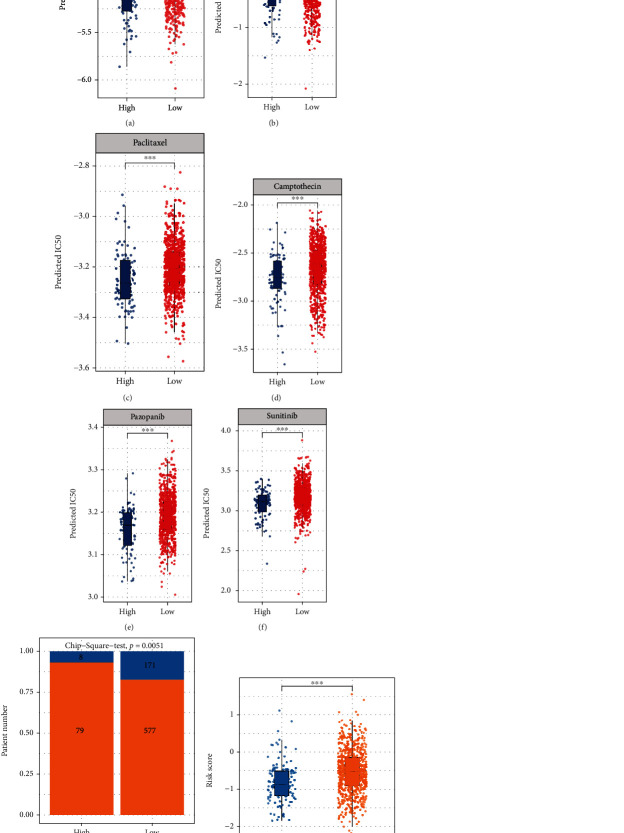
Prediction of chemotherapy and immunotherapy response. (a–f) pRRophetic approach to estimate the normalized Z-scores of IC50 for six anticancer medications: docetaxel (a), gemcitabine (b), paclitaxel (c), camptothecin (d), pazopanib (e), and sunitinib (f). (g, h). Variations in risk ratings depending on anticipated immunosuppressant treatment effects (^∗^*p* < 0.05, ^∗∗^*p* < 0.01, ^∗∗∗^*p* < 0.001).

Next, in order to determine if the model may play a role in directing the clinical treatment of breast cancer, we employed the “pRRophetic” program to predict patient sensitivity to chemotherapeutic drug therapy using the integrated Cancer Genome Project (CGP) drug database. Patients in the high-risk group in the TCGA-BRCA cohort were shown to be more responsive to chemotherapy medications (docetaxel, gemcitabine, paclitaxel, camptothecin, pazopanib, and sunitinib) than those in the low-risk group (Figures 6(a)–6(f)). In addition, we employed the TIDE online algorithm to predict the response of immune checkpoint inhibitors in TCGA-BRCA breast cancer patients. Patients in the high-risk group responded better to immune checkpoint treatment (90.1%, 79/87) than those in the low-risk group (77.1%, 577/748; *p* < 0.01). (Figure 6(g)). The risk score of patients who reacted to immunotherapy was substantially greater than that of individuals who did not respond (Figure 6(h)). The findings indicated that high-risk individuals may react more favorably to clinical chemotherapy and immunotherapy than low-risk patients.

### 3.6. Construction and Validation of Nomograms

**Figure 7:**
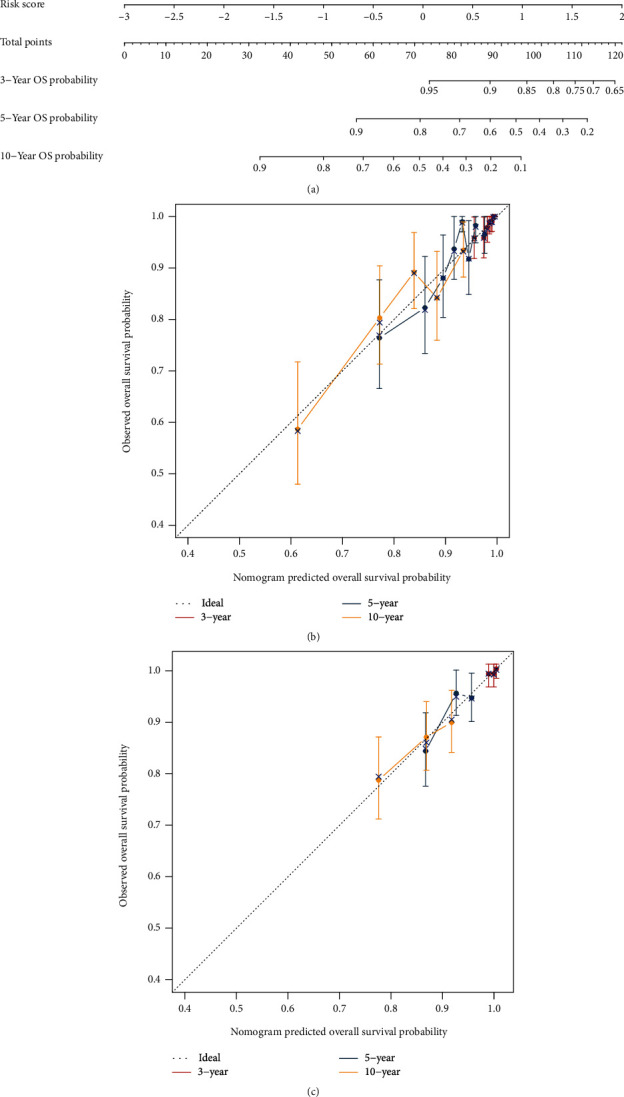
Construction and validation of a nomogram. (a) Age and risk score-based clinical line plot for predicting 3-, 5-, and 10-year total breast cancer patient survival. (b) Validation of standard curve consistencies between projected 3-, 5-, and 10-year overall survival and actual 3-, 5-, and 10-year overall survival using TCGA-BRCA cohort data. (c) Standard curves to validate the congruence between projected 3-, 5-, and 10-year overall survival and actual 3-, 5-, and 10-year overall survival using external validation data GSE20685.

According to prior research, risk score is a reliable prognostic indicator. However, it is challenging to address the specific peculiarities of clinical patients and their clinical use. We included several therapeutically relevant TCGA-BRCA cohort markers (Table 1). Analysis of the link between clinically relevant parameters, risk score, and patient prognosis using univariate and multivariate Cox regression is shown in Table 2. The prognosis of patients was connected with risk score, cancer grade (pathologic M, pathologic N, pathologic T, and tumor stage), and age, according to univariate Cox regression analysis (Table 2). Cancer grade (pathologic M, pathologicN, pathologicT, and tumor stage) had no significant in test for independence (*p* > 0.05), whereas age (HR = 2.45, *p* < 0.001) and risk score (HR = 1.03, *p* < 0.001) remained significantly (Table 2), indicating that age and risk score were independent prognostic factors. Moreover, the risk score was the most influential predictive component. Subsequently, we developed a prognostic nomogram using risk score and age to objectively estimate the 3, 5, and 10-year survival probability of patients (Figure 7(a)). The calibration curves demonstrate that TCGA-BRCA cohort data (Figure 7(b)) and external validation data GSE20685 (Figure 7(c)) are in excellent agreement with the ideal projected probability (gray dotted line) for predicting 3-year, 5-year, and 10-year survival. The findings demonstrate that this prognostic nomogram is a valid instrument for predicting OS in patients with breast cancer.

## 4. Discussion

Globally, breast cancer is the leading type of malignancy causing death in women. Significant developments in diagnostics, surgery, and anticancer drug development have been made with advances in medical technology, but effective treatment is still hampered by the metastasis and treatment resistance. Anticancer treatments have long focused on targeting tumor cells. However, recent advances in immunotherapy have shown that targeting the tumor microenvironment (TME) is a powerful tool for controlling tumor progression. Cancer-associated fibroblasts (CAFs) are the most abundant stromal cells in breast cancer, and there is growing evidence that these cells affect cancer. The exact origin of CAFs in breast cancer is not fully understood. In tumors, CAFs play an active role in the formation of TME, supporting tumor cell survival, angiogenesis, immunosuppression, and therapeutic resistance.

In this investigation, we collected a scRNA-seq data collection including 193 CAF-related marker genes from fibroblast cells. Using GO and KEGG enrichment analysis, we determined that the enriched words were associated with fibroblasts. In addition, univariate, LASSO, and multivariate Cox regression analyses allowed us to choose five key risk variables (*BGN, LUM, CCL19, CEBPD,* and *ID3*) for the construction of a signature and nomogram.

The *BGN* gene encoding biglycan, a soluble extracellular protein that belongs to the small leucine-rich proteoglycan (SLRP) family. Biglycan may perform its activity through intercellular contact, which is overexpressed in cancer stem cells [27] and may activate NF-*κ*B signaling. It binds to the extracellular matrix in the physiological context and is also expressed on the cell surface [28]. *BGN* plays an important role in various cellular processes such as cell migration, adhesion, inflammation, cell growth, regulation of autophagy, apoptosis, and regulation of matrix assembly [29]. In addition, BGN has been implicated in various tissue-specific tumorigenesis, such as pancreatic, gastric, endometrial, colon, and bladder cancers [28]. Previous studies have shown the role of *BGN* in the treatment of drug resistance and immune activity. [27, 30–32]. These suggest that *BGN* plays an important role in tumorigenesis and metastasis.

*LUM* is located on chromosome 12q21.3-q22 including a putative 18-residue signal peptide and has 338 amino acids. *LUM* core protein contains a central region rich in leucine-rich repeats, flanked by a disulfide binding region, and the central region of the molecule contains four asparagine residues capable of N-chain glycosylation [32, 33]. *LUM* is thought to be a key regulator of collagen fibrogenesis, a key process in corneal transparency [34]. *LUM* mRNA is specifically expressed in breast cancer tissues but not in normal breast tissues, suggesting that *LUM* is differentially expressed during breast tumor progression [35]. In addition, *LUM*, one of the three primary components of the corneal stroma, regulates the assembly of collagen into fibrils in diverse connective tissues. *LUM* may block or even revert the many metastatic characteristics that EMT confers to breast cancer cells [36]. These results suggest that *LUM* protein plays an important role in the growth and invasion of cancer cells.

Chemokine ligand 19 (*CCL19*) is one of the ligands of chemokine receptor 7 (*CCR7*) and plays an important role in cancer. *CCL19* has significant chemotactic action for T and B cells [37]. There is evidence that *CCL19* increases the life span of T cells within the LN. Once inside the LN, *CCL19* and *CCL21* are continually released by fibroblastic reticular cells [38, 39]. Based on the fact that CCR7 functions in the inflammatory/immune response, many strategies have been developed to exploit this axis for the treatment of cancer [40–42].

*CEBPD* is a leucine zipper (LZ) DNA-binding protein that is generally not highly expressed but can be induced by many different stimuli and is considered to be a stress response gene [43]. It is an important transcription factor regulating the expression of genes involved in immune and inflammatory responses [44, 45]. *CEBPD* has many tumor suppressor-like properties and downregulated in several types of cancer [46–49], and its expression in tumors is associated with a favorable prognosis [50, 51].

*ID3* regulates several biological processes, including cell proliferation, senescence, differentiation, apoptosis, angiogenesis, and tumor transformation. This study presented early evidence that these genes are intimately associated with the clinical manifestations and prognosis of BRCA, providing new research areas and suggestions for discovering novel gene therapy targets and producing antitumor medications.

This research has certain drawbacks. First, despite the predictability of the robustness of the features and nomogram produced in this work utilizing enormous quantities of data from the TCGA and GEO databases, they are still limited by retrospective analysis. Second, we examined the immune microenvironment landscape and molecular processes of patients at varying risk, as well as predicted the effectiveness of immunotherapy and chemotherapy; nevertheless, this study requires more experimental confirmation.

## 5. Conclusions

Based on the analysis of scRNA-seq and bulk RNA-seq data, we built and validated a cancer fibroblast-related risk signature consisting of five genes (*BGN, LUM, CCL19, CEBPD*, and *ID3*) that may be utilized as an independent prognostic indicator for breast cancer patients. In addition, this signature may suggest the vulnerability of BRCA patients to chemotherapeutic medicines (docetaxel, gemcitabine, paclitaxel, camptothecin, pazopanib, sunitinib) and immune checkpoint inhibitors, presenting BRCA patients with novel clinical uses. Ultimately, the developed signature is strong and can reliably predict the fate of BRCA patients, allowing clinicians to make more rational and viable treatment options.

## Figures and Tables

**Table 1:** Clinical information data for samples from the TCGA-BRCA cohort.

	Overall
*N*	835
Status = 1 (%)	125 (15.0)
Time (mean (SD))	1288.36 (1165.58)
Pathologic_M (%)	
M0	705 (84.4)
M1	16 (1.9)
MX	114 (13.7)
Pathologic_N (%)	
N0	391 (46.8)
N1	286 (34.3)
N2	88 (10.5)
N3	56 (6.7)
NX	14 (1.7)
Pathologic_T (%)	
T1	219 (26.2)
T2	479 (57.4)
T3	111 (13.3)
T4	25 (3.0)
TX	1 (0.1)
Tumor_stage (%)	
Stage i	149 (17.8)
Stge ii	475 (56.9)
Stage iii	179 (21.4)
Stage iv	15 (1.8)
Stage x	8 (1.0)
Not reported	9 (1.1)
Age (mean (SD))	58.16 (12.89)

**Table 2:** Univariate and multivariate Cox proportional hazards regression analysis on OS.

	Univariate analysis			Multivariate analysis		
	HR	95% CI for HR	*p* value	HR	95% CI for HR	*p* value
RiskScore	2.72	2.04-3.62	<0.001	2.45	1.54-3.89	<0.001
Pathologic_M	4.85	2.66-8.85	<0.001	0.55	0.04-6.9	0.646
Pathologic_N	1.96	1.28 3	<0.001	1.63	0.91-2.91	0.099
Pathologic_T	1.21	0.77-1.9	<0.001	1.65	0.73-3.71	0.231
Tumor_stage	1.27	0.74-2.19	<0.001	0.57	0.2-1.6	0.283
Age	1.03	1.02-1.05	<0.001	1.03	1.01-1.04	<0.001

## Data Availability

All data used to support the findings of this study are included within the article.

## Supplementary Materials

Supplementary 1 Supplementary Figure S1: two thousand highly variable genes were chosen for further study. Supplementary Figure S2: violin plot displaying the gene expression of nine markers.

Supplementary 2 Supplementary Table S1: GO enrichment findings.

Supplementary 3 Supplementary Table S2: KEGG enrichment findings.

Supplementary 4 Supplementary Table S3: list of 193 cancer-associated fibroblasts genes.
