# Supplementary material for: Development of a Cancer-Associated Fibroblast-Related Prognostic Model in Breast Cancer via Bulk and Single-Cell RNA Sequencing
Source: Biomed Res Int. 2022 Dec 2;2022:2955359. doi: 10.1155/2022/2955359 (PMC9735320; doi:10.1155/2022/2955359)
Supplement: Supplementary 1 — Supplementary Figure S1: two thousand highly variable genes were chosen for further study. Supplementary Figure S2: violin plot displaying the gene expression of nine markers. [file 2955359.f1.docx]

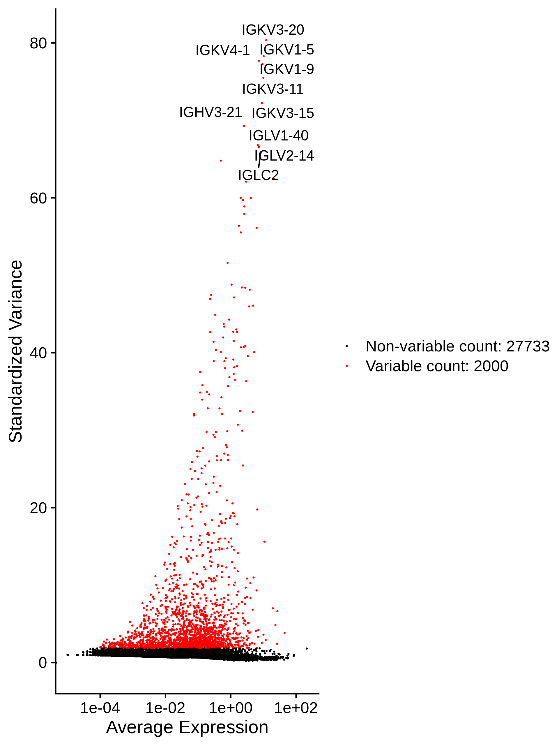


Supplemental Figure S1: Two thousand highly variable genes were chosen for further study.


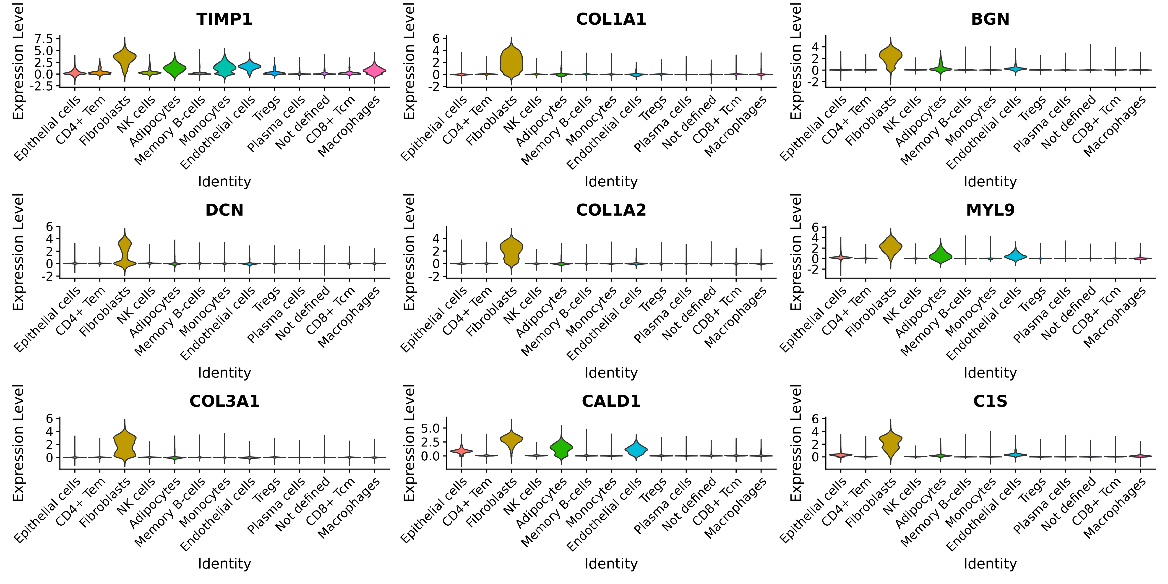


Supplemental Figure S2: Violin plot displaying the gene expression of nine markers.
