# Supplementary material for: Development of a Cancer-Associated Fibroblast-Related Prognostic Model in Breast Cancer via Bulk and Single-Cell RNA Sequencing
Source: Biomed Res Int. 2022 Dec 2;2022:2955359. doi: 10.1155/2022/2955359 (PMC9735320; doi:10.1155/2022/2955359)
Supplement: Supplementary 2 — Supplementary Table S1: GO enrichment findings. [file 2955359.f2.pdf]

Type	ID	Description	GeneRa	BgRatio	pvalue	p.adjust	qvalue	geneID	Count
BP	GO:0030198	extracellular	35/177	301/1872	3.76E-28	5.02E-25	3.75E-25	COL1A	35
BP	GO:0043062	extracellular	35/177	302/1872	4.22E-28	5.02E-25	3.75E-25	COL1A	35
BP	GO:0045229	external enca	35/177	304/1872	5.31E-28	5.02E-25	3.75E-25	COL1A	35
BP	GO:0030199	collagen fibri	17/177	61/18723	6.58E-21	4.67E-18	3.49E-18	COL1A	17
BP	GO:0052547	regulation of	27/177	461/1872	3.24E-14	1.84E-11	1.37E-11	TIMP1/	27
BP	GO:0010466	negative regu	20/177	262/1872	6.95E-13	3.29E-10	2.46E-10	TIMP1/	20
BP	GO:0042060	wound healir	24/177	422/1872	1.83E-12	7.44E-10	5.55E-10	TIMP1/	24
BP	GO:0032963	collagen met	13/177	104/1872	1.79E-11	6.35E-09	4.74E-09	COL1A	13
BP	GO:0010951	negative regu	18/177	252/1872	3.17E-11	8.98E-09	6.71E-09	TIMP1/	18
BP	GO:0061448	connective tis	18/177	252/1872	3.17E-11	8.98E-09	6.71E-09	TIMP1/	18
BP	GO:0051346	negative regu	21/177	379/1872	8.01E-11	2.07E-08	1.54E-08	TIMP1/	21
BP	GO:0045861	negative regu	20/177	351/1872	1.42E-10	3.36E-08	2.51E-08	TIMP1/	20
BP	GO:0051271	negative regu	20/177	367/1872	3.12E-10	6.81E-08	5.09E-08	TIMP1/	20
BP	GO:0052548	regulation of	21/177	432/1872	8.74E-10	1.77E-07	1.32E-07	TIMP1/	21
BP	GO:2000146	negative regu	19/177	359/1872	1.45E-09	2.74E-07	2.05E-07	TIMP1/	19
BP	GO:0031589	cell-substrate	19/177	363/1872	1.74E-09	3.09E-07	2.31E-07	COL1A	19
BP	GO:0071559	response to t	16/177	256/1872	2.90E-09	4.75E-07	3.55E-07	COL1A	16
BP	GO:0010810	regulation of	15/177	221/1872	3.02E-09	4.75E-07	3.55E-07	COL1A	15
BP	GO:0051216	cartilage dev	14/177	190/1872	3.59E-09	5.37E-07	4.01E-07	TIMP1/	14
BP	GO:0030336	negative regu	18/177	344/1872	4.80E-09	6.81E-07	5.09E-07	TIMP1/	18
BP	GO:0040013	negative regu	19/177	391/1872	5.89E-09	7.96E-07	5.94E-07	TIMP1/	19
BP	GO:0007178	transmembra	18/177	355/1872	7.85E-09	1.01E-06	7.56E-07	COL1A	18
BP	GO:0071560	cellular respo	15/177	250/1872	1.60E-08	1.98E-06	1.48E-06	COL1A	15
BP	GO:0001503	ossification	18/177	408/1872	6.60E-08	7.81E-06	5.83E-06	COL1A	18
BP	GO:0045765	regulation of	16/177	342/1872	1.67E-07	1.90E-05	1.42E-05	DCN/S	16
BP	GO:0035987	endodermal	7/177	45/18723	2.01E-07	2.14E-05	1.60E-05	COL6A	7
BP	GO:0070482	response to	16/177	347/1872	2.03E-07	2.14E-05	1.60E-05	COL1A	16
BP	GO:1901342	regulation of	16/177	348/1872	2.11E-07	2.14E-05	1.60E-05	DCN/S	16
BP	GO:0010812	negative regu	8/177	68/18723	2.48E-07	2.43E-05	1.81E-05	COL1A	8
BP	GO:0060326	cell chemota	15/177	310/1872	2.69E-07	2.51E-05	1.87E-05	CXCL9/	15
BP	GO:0014911	positive regu	7/177	47/18723	2.74E-07	2.51E-05	1.87E-05	POSTN	7
BP	GO:0050900	leukocyte mi	16/177	369/1872	4.64E-07	4.11E-05	3.07E-05	APOD/	16
BP	GO:0032964	collagen bios	7/177	51/18723	4.89E-07	4.20E-05	3.14E-05	COL1A	7
BP	GO:0007517	muscle organ	15/177	327/1872	5.30E-07	4.43E-05	3.31E-05	DCN/T	15
BP	GO:1903034	regulation of	11/177	167/1872	5.56E-07	4.51E-05	3.37E-05	SERPIN	11
BP	GO:0001706	endoderm fo	7/177	54/18723	7.30E-07	5.75E-05	4.30E-05	COL6A	7
BP	GO:0007568	aging	15/177	339/1872	8.35E-07	6.41E-05	4.79E-05	TIMP1/	15
BP	GO:0048771	tissue remod	11/177	175/1872	8.85E-07	6.44E-05	4.81E-05	TIMP1/	11
BP	GO:0045785	positive regu	17/177	437/1872	9.07E-07	6.44E-05	4.81E-05	FN1/SF	17
BP	GO:0050673	epithelial cell	17/177	437/1872	9.07E-07	6.44E-05	4.81E-05	IGFBP4	17
BP	GO:0032496	response to l	15/177	343/1872	9.67E-07	6.70E-05	5.00E-05	DCN/S	15
BP	GO:0001666	response to h	14/177	307/1872	1.38E-06	9.32E-05	6.96E-05	SOD3/I	14
BP	GO:0003012	muscle syster	17/177	452/1872	1.44E-06	9.50E-05	7.10E-05	MYL9/(	17
BP	GO:0002237	response to r	15/177	363/1872	1.96E-06	0.000124	9.27E-05	DCN/S	15
BP	GO:0014910	regulation of	8/177	89/18723	2.00E-06	0.000124	9.27E-05	POSTN	8
BP	GO:0006959	humoral imm	14/177	317/1872	2.01E-06	0.000124	9.27E-05	C1S/SE	14
BP	GO:0007160	cell-matrix a	12/177	233/1872	2.30E-06	0.000139	0.000104	COL3A	12
BP	GO:0051928	positive regu	9/177	122/1872	2.40E-06	0.00014	0.000104	THY1/(	9
BP	GO:0036293	response to	14/177	322/1872	2.41E-06	0.00014	0.000104	SOD3/I	14
BP	GO:0043567	regulation of	5/177	24/18723	2.62E-06	0.000149	0.000111	IGFBP4	5
BP	GO:0007179	transforming	11/177	198/1872	2.97E-06	0.000162	0.000121	COL1A	11
BP	GO:0031099	regeneration	11/177	198/1872	2.97E-06	0.000162	0.000121	APOD/	11
BP	GO:0050678	regulation of	15/177	381/1872	3.54E-06	0.000189	0.000141	SPARC	15
BP	GO:0014909	smooth musc	8/177	97/18723	3.85E-06	0.000202	0.000151	POSTN	8
BP	GO:0030178	negative regu	10/177	170/1872	5.10E-06	0.000253	0.000189	IGFBP4	10
BP	GO:0010712	regulation of	6/177	47/18723	5.11E-06	0.000253	0.000189	PDGFR	6
BP	GO:0061041	regulation of	9/177	134/1872	5.21E-06	0.000253	0.000189	SERPIN	9

BP	GO:0002685	regulation of	11/177	210/1872	5.22E-06	0.000253	0.000189	APOD/	11
BP	GO:1903036	positive regu	7/177	72/18723	5.26E-06	0.000253	0.000189	PLPP3/	7
BP	GO:0006936	muscle contr	14/177	347/1872	5.72E-06	0.000271	0.000202	MYL9/	14
BP	GO:0090092	regulation of	12/177	256/1872	6.06E-06	0.000282	0.000211	SFRP2/	12
BP	GO:0090090	negative regu	9/177	137/1872	6.25E-06	0.000286	0.000214	IGFBP4	9
BP	GO:0009612	response to r	11/177	216/1872	6.83E-06	0.000308	0.00023	COL1A	11
BP	GO:0060537	muscle tissue	15/177	403/1872	6.96E-06	0.000309	0.000231	DCN/IC	15
BP	GO:0007596	blood coagu	11/177	217/1872	7.14E-06	0.000309	0.000231	MYL9/	11
BP	GO:1905049	negative regu	4/177	14/18723	7.18E-06	0.000309	0.000231	TIMP1/	4
BP	GO:0001936	regulation of	10/177	179/1872	8.05E-06	0.000339	0.000254	SPARC	10
BP	GO:0097529	myeloid leuk	11/177	220/1872	8.13E-06	0.000339	0.000254	CXCL9/	11
BP	GO:0007492	endoderm de	7/177	77/18723	8.25E-06	0.00034	0.000254	COL6A	7
BP	GO:0048660	regulation of	10/177	180/1872	8.45E-06	0.000343	0.000256	APOD/	10
BP	GO:0007599	hemostasis	11/177	222/1872	8.86E-06	0.000349	0.000261	MYL9/	11
BP	GO:0050817	coagulation	11/177	222/1872	8.86E-06	0.000349	0.000261	MYL9/	11
BP	GO:0014812	muscle cell m	8/177	110/1872	9.82E-06	0.000382	0.000285	POSTN	8
BP	GO:0048659	smooth musc	10/177	184/1872	1.02E-05	0.000393	0.000294	APOD/	10
BP	GO:0016525	negative regu	9/177	149/1872	1.23E-05	0.000467	0.000349	DCN/S	9
BP	GO:2000181	negative regu	9/177	151/1872	1.37E-05	0.000513	0.000383	DCN/S	9
BP	GO:1901343	negative regu	9/177	152/1872	1.45E-05	0.000534	0.000399	DCN/S	9
BP	GO:0030111	regulation of	13/177	328/1872	1.51E-05	0.000548	0.000409	COL1A	13
BP	GO:0001935	endothelial c	10/177	193/1872	1.55E-05	0.000558	0.000417	SPARC	10
BP	GO:0002683	negative regu	15/177	434/1872	1.67E-05	0.000594	0.000444	COL3A	15
BP	GO:0071674	mononuclear	10/177	196/1872	1.78E-05	0.000622	0.000465	APOD/	10
BP	GO:0018149	peptide cross	5/177	35/18723	1.84E-05	0.000637	0.000476	BGN/D	5
BP	GO:0001704	formation of	8/177	121/1872	1.98E-05	0.000675	0.000505	COL6A	8
BP	GO:0016055	Wnt signaling	15/177	444/1872	2.18E-05	0.000737	0.000551	COL1A	15
BP	GO:0198738	cell-cell signa	15/177	446/1872	2.30E-05	0.000763	0.00057	COL1A	15
BP	GO:1904705	regulation of	7/177	90/18723	2.31E-05	0.000763	0.00057	IGFBP5	7
BP	GO:0048009	insulin-like g	5/177	37/18723	2.43E-05	0.000794	0.000593	IGFBP4	5
BP	GO:1990874	vascular asso	7/177	91/18723	2.49E-05	0.000798	0.000596	IGFBP5	7
BP	GO:0071621	granulocyte	8/177	125/1872	2.50E-05	0.000798	0.000596	CXCL9/	8
BP	GO:0050680	negative regu	9/177	164/1872	2.65E-05	0.000837	0.000625	SPARC	9
BP	GO:0022617	extracellular	6/177	63/18723	2.85E-05	0.000889	0.000664	CTSK/N	6
BP	GO:0007162	negative regu	12/177	303/1872	3.25E-05	0.001003	0.000749	COL1A	12
BP	GO:0006956	complement	8/177	130/1872	3.32E-05	0.001013	0.000756	C1S/SE	8
BP	GO:0090287	regulation of	12/177	304/1872	3.36E-05	0.001014	0.000757	DCN/S	12
BP	GO:0090101	negative regu	8/177	131/1872	3.51E-05	0.001047	0.000782	SFRP2/	8
BP	GO:0030193	regulation of	6/177	66/18723	3.72E-05	0.001101	0.000822	SERPIN	6
BP	GO:0070372	regulation of	12/177	309/1872	3.94E-05	0.001151	0.00086	FN1/C	12
BP	GO:0030449	regulation of	4/177	21/18723	4.07E-05	0.00118	0.000881	SERPIN	4
BP	GO:1900046	regulation of	6/177	68/18723	4.42E-05	0.001266	0.000946	SERPIN	6
BP	GO:0060840	artery develo	7/177	100/1872	4.58E-05	0.0013	0.000971	PDGFR	7
BP	GO:0000302	response to r	10/177	222/1872	5.14E-05	0.001445	0.001079	COL1A	10
BP	GO:0045123	cellular extra	6/177	70/18723	5.21E-05	0.001449	0.001083	THY1/	6
BP	GO:0050818	regulation of	6/177	71/18723	5.65E-05	0.001541	0.001151	SERPIN	6
BP	GO:0071230	cellular respo	6/177	71/18723	5.65E-05	0.001541	0.001151	COL1A	6
BP	GO:0048661	positive regu	7/177	104/1872	5.89E-05	0.001571	0.001173	IGFBP5	7
BP	GO:0032355	response to e	8/177	141/1872	5.92E-05	0.001571	0.001173	COL1A	8
BP	GO:0050921	positive regu	8/177	141/1872	5.92E-05	0.001571	0.001173	CCL19/	8
BP	GO:0150063	visual system	13/177	375/1872	6.03E-05	0.001584	0.001183	SERPIN	13
BP	GO:0030595	leukocyte che	10/177	230/1872	6.92E-05	0.001801	0.001345	CXCL9/	10
BP	GO:0007229	integrin-mec	7/177	107/1872	7.06E-05	0.001808	0.00135	TIMP1/	7
BP	GO:0048880	sensory syste	13/177	381/1872	7.08E-05	0.001808	0.00135	SERPIN	13
BP	GO:0048844	artery morph	6/177	74/18723	7.13E-05	0.001808	0.00135	PDGFR	6
BP	GO:0070371	ERK1 and ER	12/177	330/1872	7.40E-05	0.001859	0.001389	FN1/C	12
BP	GO:0001937	negative regu	6/177	76/18723	8.29E-05	0.002046	0.001528	SPARC	6
BP	GO:0097530	granulocyte r	8/177	148/1872	8.33E-05	0.002046	0.001528	CXCL9/	8

BP	GO:1905048	regulation of	4/177	25/18723	8.36E-05	0.002046	0.001528	TIMP1/	4
BP	GO:1903053	regulation of	5/177	48/18723	8.79E-05	0.002132	0.001593	AEBP1/	5
BP	GO:0090288	negative regu	7/177	111/1872	8.91E-05	0.002142	0.0016	DCN/S	7
BP	GO:0048251	elastic fiber a	3/177	10/18723	9.49E-05	0.002264	0.001691	MFAP4	3
BP	GO:2001236	regulation of	8/177	151/1872	9.59E-05	0.002268	0.001694	SFRP2/	8
BP	GO:1904707	positive regu	5/177	49/18723	9.72E-05	0.002279	0.001702	IGFBP5	5
BP	GO:0044344	cellular respo	7/177	113/1872	9.97E-05	0.002319	0.001732	COL1A	7
BP	GO:0071229	cellular respo	6/177	80/18723	0.00011	0.002548	0.001903	COL1A	6
BP	GO:0071675	regulation of	7/177	115/1872	0.000111	0.002548	0.001903	APOD/	7
BP	GO:0033002	muscle cell p	10/177	248/1872	0.000129	0.002897	0.002164	APOD/	10
BP	GO:0043277	apoptotic cel	5/177	52/18723	0.000129	0.002897	0.002164	MFGE8	5
BP	GO:0001867	complement	3/177	11/18723	0.00013	0.002897	0.002164	SERPIN	3
BP	GO:0009410	response to >	14/177	462/1872	0.000131	0.002913	0.002176	COL1A	14
BP	GO:0071774	response to f	7/177	120/1872	0.000145	0.003188	0.002382	COL1A	7
BP	GO:0072001	renal system	11/177	302/1872	0.000146	0.003188	0.002382	DCN/A	11
BP	GO:0060070	canonical Wr	11/177	303/1872	0.00015	0.003257	0.002433	COL1A	11
BP	GO:0060828	regulation of	10/177	253/1872	0.000152	0.003261	0.002436	COL1A	10
BP	GO:0010524	positive regu	5/177	54/18723	0.000155	0.00331	0.002472	THY1/C	5
BP	GO:0002688	regulation of	7/177	122/1872	0.000161	0.003378	0.002523	CCL19/	7
BP	GO:0051924	regulation of	10/177	255/1872	0.000162	0.003378	0.002523	THY1/C	10
BP	GO:0001558	regulation of	13/177	414/1872	0.000162	0.003378	0.002523	IGFBP7	13
BP	GO:1905517	macrophage	5/177	55/18723	0.000169	0.003455	0.002581	RARRES	5
BP	GO:0010811	positive regu	7/177	123/1872	0.000169	0.003455	0.002581	FN1/TH	7
BP	GO:0009886	post-embryo	3/177	12/18723	0.000172	0.003455	0.002581	FBN1/E	3
BP	GO:0038063	collagen-acti	3/177	12/18723	0.000172	0.003455	0.002581	COL1A	3
BP	GO:0043568	positive regu	3/177	12/18723	0.000172	0.003455	0.002581	IGFBP4	3
BP	GO:0070098	chemokine-r	6/177	88/18723	0.000187	0.00374	0.002794	CXCL9/	6
BP	GO:0090130	tissue migrat	12/177	365/1872	0.00019	0.003777	0.002822	DCN/S	12
BP	GO:0002063	chondrocyte	4/177	31/18723	0.000199	0.003917	0.002926	SFRP2/	4
BP	GO:0009615	response to v	12/177	367/1872	0.0002	0.003917	0.002926	ACTA2	12
BP	GO:0016049	cell growth	14/177	482/1872	0.000204	0.003958	0.002957	IGFBP7	14
BP	GO:0034109	homotypic ce	6/177	90/18723	0.000212	0.004087	0.003053	MYL9/I	6
BP	GO:0017015	regulation of	7/177	128/1872	0.000217	0.004152	0.003102	TGFB1I	7
BP	GO:0035904	aorta develo	5/177	58/18723	0.000218	0.004152	0.003102	PDGFR	5
BP	GO:0001654	eye developr	12/177	371/1872	0.000221	0.004164	0.003111	SERPIN	12
BP	GO:1902043	positive regu	3/177	13/18723	0.000222	0.004164	0.003111	TIMP3/	3
BP	GO:0090303	positive regu	5/177	59/18723	0.000236	0.004413	0.003297	PLPP3/	5
BP	GO:0097191	extrinsic apo	9/177	219/1872	0.000244	0.00452	0.003376	SFRP2/	9
BP	GO:1903844	regulation of	7/177	131/1872	0.00025	0.004606	0.00344	TGFB1I	7
BP	GO:0031102	neuron proje	5/177	60/18723	0.000256	0.004686	0.0035	APOD/	5
BP	GO:0002690	positive regu	6/177	94/18723	0.000268	0.004882	0.003647	CCL19/	6
BP	GO:0050920	regulation of	9/177	223/1872	0.000279	0.004998	0.003734	CCL19/	9
BP	GO:0010771	negative regu	3/177	14/18723	0.00028	0.004998	0.003734	POSTN	3
BP	GO:1900025	negative regu	3/177	14/18723	0.00028	0.004998	0.003734	POSTN	3
BP	GO:0007044	cell-substrate	6/177	95/18723	0.000284	0.005041	0.003766	APOD/	6
BP	GO:0035909	aorta morpho	4/177	34/18723	0.000287	0.005057	0.003778	PDGFR	4
BP	GO:0001101	response to a	7/177	135/1872	0.0003	0.005205	0.003888	COL1A	7
BP	GO:0002687	positive regu	7/177	135/1872	0.0003	0.005205	0.003888	THY1/C	7
BP	GO:1904035	regulation of	6/177	96/18723	0.000301	0.005205	0.003888	CD248/	6
BP	GO:1990868	response to c	6/177	97/18723	0.000318	0.005439	0.004063	CXCL9/	6
BP	GO:1990869	cellular respo	6/177	97/18723	0.000318	0.005439	0.004063	CXCL9/	6
BP	GO:0001569	branching in	4/177	35/18723	0.000322	0.005464	0.004082	SFRP2/	4
BP	GO:0045766	positive regu	8/177	181/1872	0.000332	0.005553	0.004148	SFRP2/	8
BP	GO:1904018	positive regu	8/177	181/1872	0.000332	0.005553	0.004148	SFRP2/	8
BP	GO:0043542	endothelial c	10/177	279/1872	0.000333	0.005553	0.004148	DCN/S	10
BP	GO:0048569	post-embryo	3/177	15/18723	0.000348	0.005736	0.004285	FBN1/E	3
BP	GO:0090594	inflammatory	3/177	15/18723	0.000348	0.005736	0.004285	TIMP1/	3
BP	GO:0001655	urogenital sy	11/177	338/1872	0.000384	0.006271	0.004684	DCN/A	11

BP	GO:0007369	gastrulation	8/177	185/18723	0.000384	0.006271	0.004684	COL6A	8
BP	GO:0150115	cell-substrate	6/177	101/18723	0.000396	0.006415	0.004792	APOD/	6
BP	GO:0008217	regulation of	8/177	186/18723	0.000399	0.006415	0.004792	COL1A	8
BP	GO:0072678	T cell migrati	5/177	66/18723	0.0004	0.006415	0.004792	APOD/	5
BP	GO:0072109	glomerular m	3/177	16/18723	0.000425	0.006774	0.00506	ACTA2.	3
BP	GO:0030593	neutrophil ch	6/177	103/18723	0.000439	0.006967	0.005204	CXCL9/	6
BP	GO:0048246	macrophage	4/177	38/18723	0.000443	0.006991	0.005222	RARRES	4
BP	GO:0042698	ovulation cyc	5/177	68/18723	0.00046	0.007206	0.005383	SERPIN	5
BP	GO:0001822	kidney devel	10/177	293/18723	0.000489	0.007619	0.005691	DCN/A	10
BP	GO:0007565	female pregr	8/177	193/18723	0.000509	0.007802	0.005828	TIMP1/	8
BP	GO:0002062	chondrocyte	6/177	106/18723	0.000512	0.007802	0.005828	SFRP2/	6
BP	GO:0038065	collagen-acti	3/177	17/18723	0.000512	0.007802	0.005828	COL1A	3
BP	GO:0061298	retina vascula	3/177	17/18723	0.000512	0.007802	0.005828	PDGFR	3
BP	GO:0001819	positive regu	13/177	467/18723	0.000514	0.007802	0.005828	LUM/P	13
BP	GO:1901654	response to k	8/177	194/18723	0.000527	0.007958	0.005944	IGFBP7	8
BP	GO:0051281	positive regu	4/177	40/18723	0.000541	0.008123	0.006068	THY1/C	4
BP	GO:0010952	positive regu	8/177	197/18723	0.000583	0.008712	0.006508	FN1/SF	8
BP	GO:0001953	negative regu	4/177	41/18723	0.000595	0.008795	0.00657	APOD/	4
BP	GO:1905521	regulation of	4/177	41/18723	0.000595	0.008795	0.00657	RARRES	4
BP	GO:0001667	ameboidal-ty	13/177	475/18723	0.000602	0.008796	0.00657	TIMP1/	13
BP	GO:0010631	epithelial cell	11/177	357/18723	0.000606	0.008796	0.00657	DCN/S	11
BP	GO:0006957	complement	3/177	18/18723	0.000611	0.008796	0.00657	C3/CFD	3
BP	GO:0051895	negative regu	3/177	18/18723	0.000611	0.008796	0.00657	APOD/	3
BP	GO:0150118	negative regu	3/177	18/18723	0.000611	0.008796	0.00657	APOD/	3
BP	GO:0006939	smooth musc	6/177	110/18723	0.000623	0.008936	0.006675	ACTA2.	6
BP	GO:0035924	cellular respo	5/177	73/18723	0.000638	0.009082	0.006784	DCN/P	5
BP	GO:0090132	epithelium m	11/177	360/18723	0.000649	0.009082	0.006784	DCN/S	11
BP	GO:0030574	collagen cata	4/177	42/18723	0.000653	0.009082	0.006784	CTSK/M	4
BP	GO:0032965	regulation of	4/177	42/18723	0.000653	0.009082	0.006784	PDGFR	4
BP	GO:0045773	positive regu	4/177	42/18723	0.000653	0.009082	0.006784	FN1/CX	4
BP	GO:1904037	positive regu	4/177	42/18723	0.000653	0.009082	0.006784	CD248/	4
BP	GO:0034329	cell junction a	12/177	420/18723	0.000672	0.009299	0.006946	APOD/	12
BP	GO:0007519	skeletal musc	7/177	155/18723	0.000689	0.009493	0.007091	DCN/E	7
BP	GO:0006816	calcium ion t	12/177	422/18723	0.0007	0.009599	0.007171	THY1/C	12
BP	GO:0010759	positive regu	3/177	19/18723	0.00072	0.009824	0.007339	RARRES	3
BP	GO:0048608	reproductive	12/177	424/18723	0.00073	0.009908	0.007401	DCN/S	12
BP	GO:0090596	sensory orga	9/177	256/18723	0.000756	0.010219	0.007634	THY1/F	9
BP	GO:0061458	reproductive	12/177	427/18723	0.000776	0.010435	0.007795	DCN/S	12
BP	GO:0043200	response to a	6/177	116/18723	0.000824	0.011036	0.008244	COL1A	6
BP	GO:0002920	regulation of	4/177	45/18723	0.00085	0.011274	0.008422	SERPIN	4
BP	GO:0085029	extracellular	4/177	45/18723	0.00085	0.011274	0.008422	COL1A	4
BP	GO:0072676	lymphocyte r	6/177	117/18723	0.000862	0.011336	0.008468	APOD/	6
BP	GO:0070373	negative regu	5/177	78/18723	0.000863	0.011336	0.008468	TIMP3/	5
BP	GO:0014068	positive regu	5/177	79/18723	0.000914	0.011955	0.008931	DCN/F	5
BP	GO:1901214	regulation of	10/177	319/18723	0.000939	0.012225	0.009132	SERPIN	10
BP	GO:0051607	defense resp	9/177	265/18723	0.000965	0.012447	0.009298	CXCL9/	9
BP	GO:0140546	defense resp	9/177	265/18723	0.000965	0.012447	0.009298	CXCL9/	9
BP	GO:0035455	response to i	3/177	21/18723	0.000975	0.012459	0.009307	IFITM3.	3
BP	GO:0044342	type B pancre	3/177	21/18723	0.000975	0.012459	0.009307	IGFBP4	3
BP	GO:0050878	regulation of	11/177	379/18723	0.000988	0.012576	0.009394	MYL9/C	11
BP	GO:0043618	regulation of	4/177	47/18723	0.001003	0.012706	0.009491	EGR1/J	4
BP	GO:0030512	negative regu	5/177	81/18723	0.001024	0.012886	0.009626	TGFB1I	5
BP	GO:1904019	epithelial cell	6/177	121/18723	0.001027	0.012886	0.009626	CD248/	6
BP	GO:0060538	skeletal musc	7/177	166/18723	0.001031	0.012886	0.009626	DCN/E	7
BP	GO:0050870	positive regu	8/177	216/18723	0.001061	0.013172	0.009839	THY1/C	8
BP	GO:0031960	response to c	7/177	167/18723	0.001067	0.013172	0.009839	COL1A	7
BP	GO:1990266	neutrophil m	6/177	122/18723	0.001072	0.013172	0.009839	CXCL9/	6
BP	GO:1902903	regulation of	11/177	383/18723	0.001075	0.013172	0.009839	AEBP1/	11

BP	GO:0060395	SMAD protei	5/177	82/18723	0.001082	0.013172	0.009839	FOS/VI	5
BP	GO:0002686	negative regu	4/177	48/18723	0.001086	0.013172	0.009839	APOD/	4
BP	GO:2001238	positive regu	4/177	48/18723	0.001086	0.013172	0.009839	TIMP3/	4
BP	GO:0014706	striated musc	11/177	384/1872	0.001098	0.013252	0.009899	DCN/P	11
BP	GO:0010975	regulation of	12/177	445/1872	0.001107	0.013252	0.009899	SERPIN	12
BP	GO:0030168	platelet activ	6/177	123/1872	0.001118	0.013252	0.009899	MYL9/	6
BP	GO:0055093	response to h	3/177	22/18723	0.001121	0.013252	0.009899	COL1A	3
BP	GO:0060343	trabecula for	3/177	22/18723	0.001121	0.013252	0.009899	COL1A	3
BP	GO:0090280	positive regu	3/177	22/18723	0.001121	0.013252	0.009899	PDGFR	3
BP	GO:0030195	negative regu	4/177	49/18723	0.001174	0.013598	0.010158	SERPIN	4
BP	GO:0038084	vascular endo	4/177	49/18723	0.001174	0.013598	0.010158	DCN/P	4
BP	GO:0052372	modulation b	4/177	49/18723	0.001174	0.013598	0.010158	IFITM3/	4
BP	GO:1902041	regulation of	4/177	49/18723	0.001174	0.013598	0.010158	SFRP2/	4
BP	GO:1904752	regulation of	4/177	49/18723	0.001174	0.013598	0.010158	IGFBP5	4
BP	GO:0044706	multi-multice	8/177	220/1872	0.001193	0.013767	0.010284	TIMP1/	8
BP	GO:0045445	myoblast diff	5/177	84/18723	0.001206	0.013853	0.010348	CXCL9/	5
BP	GO:0043270	positive regu	9/177	275/1872	0.001249	0.014298	0.01068	THY1/	9
BP	GO:1900047	negative regu	4/177	50/18723	0.001267	0.014379	0.010741	SERPIN	4
BP	GO:1904738	vascular asso	4/177	50/18723	0.001267	0.014379	0.010741	IGFBP5	4
BP	GO:0009314	response to r	12/177	456/1872	0.001362	0.015306	0.011433	TIMP1/	12
BP	GO:0010656	negative regu	4/177	51/18723	0.001364	0.015306	0.011433	SFRP2/	4
BP	GO:0071622	regulation of	4/177	51/18723	0.001364	0.015306	0.011433	CCL19/	4
BP	GO:0001952	regulation of	6/177	128/1872	0.001373	0.015344	0.011462	APOD/	6
BP	GO:0048041	focal adhesic	5/177	87/18723	0.001411	0.015699	0.011727	APOD/	5
BP	GO:0032970	regulation of	11/177	397/1872	0.001433	0.015883	0.011865	PDGFR	11
BP	GO:0051043	regulation of	3/177	24/18723	0.001453	0.015979	0.011936	TIMP1/	3
BP	GO:2000353	positive regu	3/177	24/18723	0.001453	0.015979	0.011936	CD248/	3
BP	GO:0048545	response to s	10/177	339/1872	0.001481	0.016228	0.012122	COL1A	10
BP	GO:0001649	osteoblast di	8/177	229/1872	0.001539	0.0168	0.01255	COL1A	8
BP	GO:0022409	positive regu	9/177	284/1872	0.00156	0.016938	0.012652	THY1/	9
BP	GO:0007566	embryo impl	4/177	53/18723	0.001576	0.016938	0.012652	TIMP1/	4
BP	GO:0043620	regulation of	4/177	53/18723	0.001576	0.016938	0.012652	EGR1/J	4
BP	GO:0050819	negative regu	4/177	53/18723	0.001576	0.016938	0.012652	SERPIN	4
BP	GO:0003094	glomerular fi	3/177	25/18723	0.001639	0.017282	0.01291	SULF1/	3
BP	GO:0072012	glomerulus v	3/177	25/18723	0.001639	0.017282	0.01291	ACTA2	3
BP	GO:1903901	negative regu	3/177	25/18723	0.001639	0.017282	0.01291	IFITM3/	3
BP	GO:1904754	positive regu	3/177	25/18723	0.001639	0.017282	0.01291	IGFBP5	3
BP	GO:0010721	negative regu	7/177	180/1872	0.001644	0.017282	0.01291	THY1/F	7
BP	GO:0043409	negative regu	7/177	180/1872	0.001644	0.017282	0.01291	SFRP2/	7
BP	GO:0097205	renal filtratio	3/177	26/18723	0.00184	0.019152	0.014307	SULF1/	3
BP	GO:1905523	positive regu	3/177	26/18723	0.00184	0.019152	0.014307	RARRES	3
BP	GO:0060485	mesenchyme	9/177	291/1872	0.001842	0.019152	0.014307	COL1A	9
BP	GO:0055001	muscle cell d	7/177	184/1872	0.001863	0.019299	0.014416	PDGFR	7
BP	GO:0051591	response to c	5/177	93/18723	0.001896	0.019567	0.014617	COL1A	5
BP	GO:1904645	response to a	4/177	56/18723	0.001933	0.01988	0.01485	MMP2/	4
BP	GO:0071478	cellular respo	7/177	186/1872	0.001981	0.020264	0.015137	TIMP1/	7
BP	GO:1903035	negative regu	5/177	94/18723	0.001987	0.020264	0.015137	SERPIN	5
BP	GO:0043434	response to p	11/177	414/1872	0.001992	0.020264	0.015137	TIMP1/	11
BP	GO:1903039	positive regu	8/177	239/1872	0.002012	0.020392	0.015233	THY1/	8
BP	GO:0010758	regulation of	3/177	27/18723	0.002056	0.020546	0.015348	RARRES	3
BP	GO:0032967	positive regu	3/177	27/18723	0.002056	0.020546	0.015348	PDGFR	3
BP	GO:0061437	renal system	3/177	27/18723	0.002056	0.020546	0.015348	ACTA2	3
BP	GO:0061440	kidney vascu	3/177	27/18723	0.002056	0.020546	0.015348	ACTA2	3
BP	GO:1900024	regulation of	4/177	57/18723	0.002064	0.020554	0.015354	POSTN	4
BP	GO:0045598	regulation of	6/177	139/1872	0.002088	0.020724	0.015481	SFRP2/	6
BP	GO:2001233	regulation of	10/177	356/1872	0.002121	0.02097	0.015665	SFRP2/	10
BP	GO:0003073	regulation of	5/177	96/18723	0.00218	0.021408	0.015992	POSTN	5
BP	GO:0030510	regulation of	5/177	96/18723	0.00218	0.021408	0.015992	SFRP2/	5

BP	GO:0032956	regulation of	10/177	358/1872	0.002209	0.021615	0.016146	PDGFR	10
BP	GO:0034341	response to i	6/177	141/1872	0.002244	0.021736	0.016237	IFITM3/	6
BP	GO:0035296	regulation of	6/177	141/1872	0.002244	0.021736	0.016237	SOD3/	6
BP	GO:0097746	blood vessel	6/177	141/1872	0.002244	0.021736	0.016237	SOD3/	6
BP	GO:1901216	positive regu	5/177	97/18723	0.002281	0.02178	0.016269	EGR1/F	5
BP	GO:2001237	negative regu	5/177	97/18723	0.002281	0.02178	0.016269	SFRP2/	5
BP	GO:0010575	positive regu	3/177	28/18723	0.002287	0.02178	0.016269	C3/CYF	3
BP	GO:0010714	positive regu	3/177	28/18723	0.002287	0.02178	0.016269	PDGFR	3
BP	GO:0060325	face morpho	3/177	28/18723	0.002287	0.02178	0.016269	COL1A	3
BP	GO:0035150	regulation of	6/177	142/1872	0.002325	0.022068	0.016485	SOD3/	6
BP	GO:0001836	release of cyt	4/177	59/18723	0.002344	0.022121	0.016525	JUN/LN	4
BP	GO:0070997	neuron death	10/177	361/1872	0.002346	0.022121	0.016525	SERPIN	10
BP	GO:0032103	positive regu	11/177	427/1872	0.00253	0.02378	0.017764	CCL19/	11
BP	GO:2000401	regulation of	4/177	61/18723	0.002648	0.024806	0.01853	APOD/	4
BP	GO:0022604	regulation of	9/177	309/1872	0.002759	0.025755	0.019239	SPARC/	9
BP	GO:0036296	response to i	3/177	30/18723	0.002795	0.02601	0.01943	COL1A	3
BP	GO:0032835	glomerulus d	4/177	62/18723	0.00281	0.026065	0.019471	ACTA2/	4
BP	GO:0010522	regulation of	5/177	102/1872	0.002839	0.026084	0.019485	THY1/C	5
BP	GO:0007159	leukocyte cel	10/177	371/1872	0.002855	0.026084	0.019485	THY1/C	10
BP	GO:0051384	response to g	6/177	148/1872	0.002858	0.026084	0.019485	IGFBP7	6
BP	GO:0009743	response to c	8/177	253/1872	0.002858	0.026084	0.019485	COL6A	8
BP	GO:0034612	response to t	8/177	253/1872	0.002858	0.026084	0.019485	COL1A	8
BP	GO:2000242	negative regu	4/177	63/18723	0.002979	0.027099	0.020243	TIMP1/	4
BP	GO:0071711	basement me	3/177	31/18723	0.003073	0.027856	0.020808	MMP1	3
BP	GO:0002253	activation of	10/177	375/1872	0.003082	0.027856	0.020808	C1S/SE	10
BP	GO:0046579	positive regu	4/177	64/18723	0.003155	0.02835	0.021178	COL3A	4
BP	GO:0048592	eye morphog	6/177	151/1872	0.003157	0.02835	0.021178	THY1/C	6
BP	GO:0030509	BMP signalin	6/177	152/1872	0.003261	0.029197	0.02181	SFRP2/	6
BP	GO:0022411	cellular comp	11/177	443/1872	0.003349	0.029593	0.022106	CTSK/C	11
BP	GO:0010667	negative regu	3/177	32/18723	0.003368	0.029593	0.022106	SFRP2/	3
BP	GO:0035767	endothelial c	3/177	32/18723	0.003368	0.029593	0.022106	SMOC2	3
BP	GO:0060323	head morpho	3/177	32/18723	0.003368	0.029593	0.022106	COL1A	3
BP	GO:0061037	negative regu	3/177	32/18723	0.003368	0.029593	0.022106	CTSK/F	3
BP	GO:1901889	negative regu	3/177	32/18723	0.003368	0.029593	0.022106	APOD/	3
BP	GO:0007204	positive regu	9/177	319/1872	0.003406	0.029767	0.022236	THY1/C	9
BP	GO:0060348	bone develop	7/177	205/1872	0.003409	0.029767	0.022236	COL1A	7
BP	GO:0006979	response to c	11/177	446/1872	0.003524	0.030237	0.022587	COL1A	11
BP	GO:0045600	positive regu	4/177	66/18723	0.003527	0.030237	0.022587	SFRP2/	4
BP	GO:0050766	positive regu	4/177	66/18723	0.003527	0.030237	0.022587	MFGE8	4
BP	GO:0050918	positive chen	4/177	66/18723	0.003527	0.030237	0.022587	CXCL1	4
BP	GO:0051893	regulation of	4/177	66/18723	0.003527	0.030237	0.022587	APOD/	4
BP	GO:0090109	regulation of	4/177	66/18723	0.003527	0.030237	0.022587	APOD/	4
BP	GO:0034614	cellular respo	6/177	155/1872	0.00359	0.030605	0.022862	SOD3/T	6
BP	GO:0003018	vascular proc	8/177	263/1872	0.003617	0.030605	0.022862	SOD3/	8
BP	GO:0043588	skin develop	8/177	263/1872	0.003617	0.030605	0.022862	COL1A	8
BP	GO:0043010	camera-type	9/177	322/1872	0.003621	0.030605	0.022862	SERPIN	9
BP	GO:0034446	substrate adh	5/177	108/1872	0.003632	0.030605	0.022862	FN1/PC	5
BP	GO:0022407	regulation of	11/177	448/1872	0.003644	0.030605	0.022862	THY1/C	11
BP	GO:0042692	muscle cell d	10/177	384/1872	0.003645	0.030605	0.022862	CXCL9/	10
BP	GO:0035025	positive regu	3/177	33/18723	0.003679	0.030622	0.022875	COL3A	3
BP	GO:0048048	embryonic ey	3/177	33/18723	0.003679	0.030622	0.022875	FBN1/E	3
BP	GO:0070570	regulation of	3/177	33/18723	0.003679	0.030622	0.022875	THY1/F	3
BP	GO:0035914	skeletal musc	4/177	67/18723	0.003723	0.030806	0.023012	EGR1/F	4
BP	GO:0070527	platelet aggr	4/177	67/18723	0.003723	0.030806	0.023012	MYL9/F	4
BP	GO:0043281	regulation of	7/177	209/1872	0.00379	0.030938	0.023111	SFRP2/	7
BP	GO:0061299	retina vascula	2/177	10/18723	0.003805	0.030938	0.023111	CYP1B1	2
BP	GO:0072203	cell proliferat	2/177	10/18723	0.003805	0.030938	0.023111	PDGFR	2
BP	GO:0072378	blood coagu	2/177	10/18723	0.003805	0.030938	0.023111	FN1/FE	2

BP	GO:0097278	complement	2/177	10/18723	0.003805	0.030938	0.023111	C3/CFH	2
BP	GO:2000425	regulation of	2/177	10/18723	0.003805	0.030938	0.023111	C3/CCL	2
BP	GO:0051017	actin filamen	6/177	157/1872	0.003823	0.030999	0.023156	CALD1	6
BP	GO:0046686	response to	4/177	68/18723	0.003927	0.031667	0.023656	SPARC	4
BP	GO:0009408	response to	5/177	110/1872	0.003928	0.031667	0.023656	IGFBP7	5
BP	GO:0051235	maintenance	9/177	327/1872	0.004004	0.032193	0.024049	THY1/	9
BP	GO:0014066	regulation of	5/177	111/1872	0.004082	0.032727	0.024447	DCN/F	5
BP	GO:0043523	regulation of	7/177	212/1872	0.004097	0.03275	0.024464	CCL2/	7
BP	GO:0060402	calcium ion t	6/177	160/1872	0.004193	0.033424	0.024968	THY1/	6
BP	GO:0051651	maintenance	7/177	214/1872	0.004311	0.034065	0.025447	THY1/	7
BP	GO:0061572	actin filamen	6/177	161/1872	0.004322	0.034065	0.025447	CALD1	6
BP	GO:0019216	regulation of	9/177	331/1872	0.004333	0.034065	0.025447	APOD/	9
BP	GO:0071214	cellular respo	9/177	331/1872	0.004333	0.034065	0.025447	TIMP1/	9
BP	GO:0104004	cellular respo	9/177	331/1872	0.004333	0.034065	0.025447	TIMP1/	9
BP	GO:0010664	negative regu	3/177	35/18723	0.004353	0.034128	0.025494	SFRP2/	3
BP	GO:0071347	cellular respo	5/177	113/1872	0.004404	0.034431	0.02572	CCL19/	5
BP	GO:0007601	visual percep	7/177	215/1872	0.004422	0.034474	0.025752	COL1A	7
BP	GO:0150116	regulation of	4/177	71/18723	0.004584	0.035161	0.026266	APOD/	4
BP	GO:0002604	regulation of	2/177	11/18723	0.004621	0.035161	0.026266	CCL19/	2
BP	GO:0014820	tonic smooth	2/177	11/18723	0.004621	0.035161	0.026266	MYLK/	2
BP	GO:0043589	skin morpho	2/177	11/18723	0.004621	0.035161	0.026266	COL1A	2
BP	GO:0071492	cellular respo	2/177	11/18723	0.004621	0.035161	0.026266	TIMP1/	2
BP	GO:0072110	glomerular m	2/177	11/18723	0.004621	0.035161	0.026266	PDGFR	2
BP	GO:0097048	dendritic cell	2/177	11/18723	0.004621	0.035161	0.026266	CCL19/	2
BP	GO:2000343	positive regu	2/177	11/18723	0.004621	0.035161	0.026266	POSTN	2
BP	GO:2000668	regulation of	2/177	11/18723	0.004621	0.035161	0.026266	CCL19/	2
BP	GO:0002691	regulation of	3/177	36/18723	0.004716	0.035789	0.026734	THY1/	3
BP	GO:0051209	release of se	5/177	115/1872	0.004743	0.035898	0.026816	THY1/	5
BP	GO:1903037	regulation of	9/177	336/1872	0.004773	0.035988	0.026883	THY1/	9
BP	GO:0033627	cell adhesion	4/177	72/18723	0.004819	0.035988	0.026883	SFRP2/	4
BP	GO:0042246	tissue regene	4/177	72/18723	0.004819	0.035988	0.026883	APOD/	4
BP	GO:0043903	regulation of	4/177	72/18723	0.004819	0.035988	0.026883	IFITM3	4
BP	GO:0051057	positive regu	4/177	72/18723	0.004819	0.035988	0.026883	COL3A	4
BP	GO:0062197	cellular respo	9/177	337/1872	0.004865	0.036008	0.026898	SOD3/	9
BP	GO:0071772	response to	6/177	165/1872	0.004867	0.036008	0.026898	SFRP2/	6
BP	GO:0071773	cellular respo	6/177	165/1872	0.004867	0.036008	0.026898	SFRP2/	6
BP	GO:0034764	positive regu	7/177	219/1872	0.004885	0.036008	0.026898	THY1/	7
BP	GO:0050953	sensory perc	7/177	219/1872	0.004885	0.036008	0.026898	COL1A	7
BP	GO:0051283	negative regu	5/177	116/1872	0.00492	0.036173	0.027022	THY1/	5
BP	GO:0030307	positive regu	6/177	166/1872	0.005011	0.036745	0.027449	FN1/SF	6
BP	GO:0061035	regulation of	4/177	73/18723	0.005061	0.036924	0.027582	CTSK/F	4
BP	GO:1904427	positive regu	4/177	73/18723	0.005061	0.036924	0.027582	THY1/	4
BP	GO:0032570	response to	3/177	37/18723	0.005097	0.036997	0.027637	FOS/C	3
BP	GO:0040036	regulation of	3/177	37/18723	0.005097	0.036997	0.027637	SULF1/	3
BP	GO:0008406	gonad devel	7/177	221/1872	0.00513	0.037138	0.027743	SFRP2/	7
BP	GO:0022612	gland morph	5/177	118/1872	0.005287	0.038082	0.028447	IGFBP5	5
BP	GO:0051282	regulation of	5/177	118/1872	0.005287	0.038082	0.028447	THY1/	5
BP	GO:0019221	cytokine-me	11/177	472/1872	0.005366	0.038474	0.02874	CXCL9/	11
BP	GO:0010959	regulation of	10/177	406/1872	0.005368	0.038474	0.02874	THY1/	10
BP	GO:0007614	short-term m	2/177	12/18723	0.005511	0.038715	0.02892	SERPIN	2
BP	GO:0010642	negative regu	2/177	12/18723	0.005511	0.038715	0.02892	APOD/	2
BP	GO:0032908	regulation of	2/177	12/18723	0.005511	0.038715	0.02892	LUM/T	2
BP	GO:0045916	negative regu	2/177	12/18723	0.005511	0.038715	0.02892	SERPIN	2
BP	GO:0060394	negative regu	2/177	12/18723	0.005511	0.038715	0.02892	LRP1/E	2
BP	GO:0061430	bone trabecu	2/177	12/18723	0.005511	0.038715	0.02892	COL1A	2
BP	GO:0072376	protein activ	2/177	12/18723	0.005511	0.038715	0.02892	FN1/FE	2
BP	GO:0090177	establishmen	2/177	12/18723	0.005511	0.038715	0.02892	SFRP2/	2
BP	GO:0048662	negative regu	4/177	75/18723	0.00557	0.039032	0.029157	APOD/	4

BP	GO:0051146	striated musc	8/177	283/1872:	0.005594	0.039103	0.02921	CXCL9/	8
BP	GO:0034284	response to r	7/177	225/1872:	0.005647	0.039377	0.029415	COL6A	7
BP	GO:0048675	axon extensic	5/177	120/1872:	0.005673	0.03946	0.029477	FN1/C>	5
BP	GO:0003206	cardiac cham	5/177	121/1872:	0.005873	0.040753	0.030443	SFRP2/	5
BP	GO:0010463	mesenchyma	3/177	39/18723	0.005913	0.040878	0.030536	PRRX1/	3
BP	GO:0045137	development	7/177	227/1872:	0.00592	0.040878	0.030536	SFRP2/	7
BP	GO:1990138	neuron proje	6/177	172/1872:	0.00594	0.040919	0.030566	FN1/PC	6
BP	GO:0043410	positive regu	11/177	480/1872:	0.006065	0.041567	0.031051	IGFBP4	11
BP	GO:0019730	antimicrobial	5/177	122/1872:	0.006078	0.041567	0.031051	CXCL9/	5
BP	GO:0051208	sequestering	5/177	122/1872:	0.006078	0.041567	0.031051	THY1/C	5
BP	GO:0071356	cellular respc	7/177	229/1872:	0.006203	0.042315	0.03161	COL1A	7
BP	GO:0007584	response to r	6/177	174/1872:	0.006276	0.042716	0.031909	COL1A	6
BP	GO:0045777	positive regu	3/177	40/18723	0.006349	0.043003	0.032123	RARRE!	3
BP	GO:0071276	cellular respc	3/177	40/18723	0.006349	0.043003	0.032123	FOS/JU	3
BP	GO:0043154	negative regu	4/177	78/18723	0.006396	0.043021	0.032137	SFRP2/	4
BP	GO:0061045	negative regu	4/177	78/18723	0.006396	0.043021	0.032137	SERPIN	4
BP	GO:0001768	establishmen	2/177	13/18723	0.006473	0.043021	0.032137	CCL19/	2
BP	GO:0014745	negative regu	2/177	13/18723	0.006473	0.043021	0.032137	IGFBP5	2
BP	GO:0032905	transforming	2/177	13/18723	0.006473	0.043021	0.032137	LUM/T	2
BP	GO:0036462	TRAIL-activat	2/177	13/18723	0.006473	0.043021	0.032137	TIMP3/	2
BP	GO:1903054	negative regu	2/177	13/18723	0.006473	0.043021	0.032137	CST3/E	2
BP	GO:2001198	regulation of	2/177	13/18723	0.006473	0.043021	0.032137	TMEM1	2
BP	GO:0051480	regulation of	9/177	353/1872:	0.006536	0.043336	0.032372	THY1/C	9
BP	GO:0072503	cellular divale	11/177	486/1872:	0.006635	0.043837	0.032747	THY1/C	11
BP	GO:0010594	regulation of	7/177	232/1872:	0.006645	0.043837	0.032747	DCN/S	7
BP	GO:0006029	proteoglycan	4/177	79/18723	0.006688	0.043837	0.032747	BGN/D	4
BP	GO:0050772	positive regu	4/177	79/18723	0.006688	0.043837	0.032747	FN1/C>	4
BP	GO:0051279	regulation of	4/177	79/18723	0.006688	0.043837	0.032747	THY1/C	4
BP	GO:0010632	regulation of	8/177	292/1872:	0.006715	0.043909	0.0328	DCN/S	8
BP	GO:0050867	positive regu	10/177	420/1872:	0.006761	0.044111	0.032951	THY1/C	10
BP	GO:0071634	regulation of	3/177	41/18723	0.006803	0.044202	0.033019	LUM/FI	3
BP	GO:0030324	lung develop	6/177	177/1872:	0.006806	0.044202	0.033019	SPARC,	6
BP	GO:2001235	positive regu	5/177	126/1872:	0.00695	0.044849	0.033502	TIMP3/	5
BP	GO:0048588	development	7/177	234/1872:	0.006953	0.044849	0.033502	FN1/PC	7
BP	GO:0060560	development	7/177	234/1872:	0.006953	0.044849	0.033502	FN1/SF	7
BP	GO:0048145	regulation of	4/177	80/18723	0.006989	0.044881	0.033526	FN1/PD	4
BP	GO:0009266	response to t	6/177	178/1872:	0.00699	0.044881	0.033526	IGFBP7	6
BP	GO:2000116	regulation of	7/177	235/1872:	0.007111	0.045557	0.034031	SFRP2/	7
BP	GO:0030048	actin filamen	5/177	127/1872:	0.00718	0.045889	0.034279	GSN/TI	5
BP	GO:1901653	cellular respc	9/177	359/1872:	0.007264	0.045889	0.034279	RARRE!	9
BP	GO:0048762	mesenchyma	7/177	236/1872:	0.007272	0.045889	0.034279	COL1A	7
BP	GO:0014912	negative regu	3/177	42/18723	0.007276	0.045889	0.034279	IGFBP5	3
BP	GO:0046596	regulation of	3/177	42/18723	0.007276	0.045889	0.034279	IFITM3,	3
BP	GO:1902895	positive regu	3/177	42/18723	0.007276	0.045889	0.034279	EGR1/F	3
BP	GO:2000404	regulation of	3/177	42/18723	0.007276	0.045889	0.034279	APOD/	3
BP	GO:0048144	fibroblast prc	4/177	81/18723	0.007299	0.045933	0.034312	FN1/PD	4
BP	GO:0032612	interleukin-1	5/177	128/1872:	0.007416	0.046404	0.034664	CCL19/	5
BP	GO:0032652	regulation of	5/177	128/1872:	0.007416	0.046404	0.034664	CCL19/	5
BP	GO:0001767	establishmen	2/177	14/18723	0.007505	0.046404	0.034664	CCL19/	2
BP	GO:0003214	cardiac left v	2/177	14/18723	0.007505	0.046404	0.034664	SFRP2/	2
BP	GO:0010838	positive regu	2/177	14/18723	0.007505	0.046404	0.034664	MDK/F	2
BP	GO:0035791	platelet-deriv	2/177	14/18723	0.007505	0.046404	0.034664	PDGFR	2
BP	GO:0050930	induction of	2/177	14/18723	0.007505	0.046404	0.034664	CXCL1:	2
BP	GO:0070141	response to u	2/177	14/18723	0.007505	0.046404	0.034664	TIMP1/	2
BP	GO:0048511	rhythmic pro	8/177	298/1872:	0.007551	0.046454	0.034701	SERPIN	8
BP	GO:0048568	embryonic or	10/177	427/1872:	0.007555	0.046454	0.034701	RARRE!	10
BP	GO:0030323	respiratory tu	6/177	181/1872:	0.007562	0.046454	0.034701	SPARC,	6
BP	GO:0008543	fibroblast grc	4/177	82/18723	0.007618	0.046596	0.034807	SULF1/	4

BP	GO:0008625	extrinsic apo	4/177	82/18723	0.007618	0.046596	0.034807	SFRP2/	4
BP	GO:0060401	cytosolic calc	6/177	182/1872	0.00776	0.046908	0.03504	THY1/C	6
BP	GO:0061138	morphogene	6/177	182/1872	0.00776	0.046908	0.03504	SFRP2/	6
BP	GO:0006509	membrane p	3/177	43/18723	0.007768	0.046908	0.03504	TIMP1/	3
BP	GO:0010171	body morph	3/177	43/18723	0.007768	0.046908	0.03504	COL1A	3
BP	GO:0061383	trabecula mo	3/177	43/18723	0.007768	0.046908	0.03504	COL1A	3
BP	GO:0071604	transforming	3/177	43/18723	0.007768	0.046908	0.03504	LUM/FI	3
BP	GO:0048678	response to	4/177	83/18723	0.007946	0.047778	0.03569	APOD/	4
BP	GO:0051899	membrane d	4/177	83/18723	0.007946	0.047778	0.03569	DCN/JU	4
BP	GO:0046683	response to	5/177	131/1872	0.008157	0.048941	0.03656	COL1A	5
BP	GO:0031295	T cell costim	3/177	44/18723	0.00828	0.049282	0.036814	CCL19/	3
BP	GO:0060324	face develop	3/177	44/18723	0.00828	0.049282	0.036814	COL1A	3
BP	GO:0010660	regulation of	4/177	84/18723	0.008283	0.049282	0.036814	SFRP2/	4
BP	GO:0042310	vasoconstrict	4/177	84/18723	0.008283	0.049282	0.036814	ACTA2	4
BP	GO:0045667	regulation of	5/177	132/1872	0.008415	0.049961	0.037321	SFRP2/	5
BP	GO:0043123	positive regu	6/177	186/1872	0.00859	0.050256	0.037542	CCL19/	6
BP	GO:0002468	dendritic cell	2/177	15/18723	0.008606	0.050256	0.037542	CCL19/	2
BP	GO:0010713	negative regu	2/177	15/18723	0.008606	0.050256	0.037542	CST3/E	2
BP	GO:0042249	establishmen	2/177	15/18723	0.008606	0.050256	0.037542	SFRP2/	2
BP	GO:0060026	convergent e	2/177	15/18723	0.008606	0.050256	0.037542	SFRP2/	2
BP	GO:0072224	metanephric	2/177	15/18723	0.008606	0.050256	0.037542	PDGFR	2
BP	GO:1901741	positive regu	2/177	15/18723	0.008606	0.050256	0.037542	CXCL9/	2
BP	GO:2000402	negative regu	2/177	15/18723	0.008606	0.050256	0.037542	APOD/	2
BP	GO:0001656	metanephros	4/177	85/18723	0.008629	0.050288	0.037565	PDGFR	4
BP	GO:1904646	cellular respo	3/177	45/18723	0.00881	0.051237	0.038274	LRP1/V	3
BP	GO:0035023	regulation of	4/177	86/18723	0.008985	0.052039	0.038874	COL3A	4
BP	GO:2000117	negative regu	4/177	86/18723	0.008985	0.052039	0.038874	SFRP2/	4
BP	GO:0031668	cellular respo	7/177	246/1872	0.009028	0.052078	0.038903	COL1A	7
BP	GO:0051402	neuron apop	7/177	246/1872	0.009028	0.052078	0.038903	CCL2/N	7
BP	GO:0045862	positive regu	9/177	372/1872	0.009056	0.052133	0.038944	FN1/SF	9
BP	GO:0010038	response to	9/177	373/1872	0.009207	0.052893	0.039511	SOD3/S	9
BP	GO:0046578	regulation of	6/177	189/1872	0.009253	0.053053	0.039631	COL3A	6
BP	GO:1901879	regulation of	4/177	87/18723	0.00935	0.053237	0.039768	GSN/M	4
BP	GO:0001974	blood vessel	3/177	46/18723	0.009361	0.053237	0.039768	BGN/C	3
BP	GO:0031294	lymphocyte c	3/177	46/18723	0.009361	0.053237	0.039768	CCL19/	3
BP	GO:0043462	regulation of	3/177	46/18723	0.009361	0.053237	0.039768	TPM2/T	3
BP	GO:0007015	actin filamen	10/177	442/1872	0.009499	0.053919	0.040278	CALD1	10
BP	GO:0045926	negative regu	7/177	249/1872	0.009611	0.054445	0.040671	SFRP2/	7
BP	GO:0002693	positive regu	2/177	16/18723	0.009775	0.054688	0.040853	THY1/N	2
BP	GO:0002921	negative regu	2/177	16/18723	0.009775	0.054688	0.040853	SERPIN	2
BP	GO:0003084	positive regu	2/177	16/18723	0.009775	0.054688	0.040853	RARRE	2
BP	GO:0044849	estrous cycle	2/177	16/18723	0.009775	0.054688	0.040853	EGR1/N	2
BP	GO:0051014	actin filamen	2/177	16/18723	0.009775	0.054688	0.040853	GSN/D	2
BP	GO:0007266	Rho protein	5/177	137/1872	0.009789	0.054688	0.040853	COL1A	5
BP	GO:0010977	negative regu	5/177	137/1872	0.009789	0.054688	0.040853	THY1/L	5
BP	GO:0048483	autonomic n	3/177	47/18723	0.00993	0.055152	0.041198	FN1/ED	3
BP	GO:0090279	regulation of	3/177	47/18723	0.00993	0.055152	0.041198	PDGFR	3
BP	GO:0097028	dendritic cell	3/177	47/18723	0.00993	0.055152	0.041198	CCL19/	3
BP	GO:0010657	muscle cell a	4/177	89/18723	0.010109	0.056032	0.041856	SFRP2/	4
BP	GO:0090257	regulation of	7/177	252/1872	0.010222	0.056547	0.042241	MYL9/I	7
BP	GO:0006874	cellular calci	10/177	448/1872	0.010376	0.057291	0.042797	THY1/C	10
BP	GO:0097305	response to	7/177	253/1872	0.010431	0.057482	0.042939	IGFBP7	7
BP	GO:0048146	positive regu	3/177	48/18723	0.01052	0.05786	0.043222	FN1/PD	3
BP	GO:0001763	morphogene	6/177	196/1872	0.010941	0.059945	0.044779	SFRP2/	6
BP	GO:0006006	glucose meta	6/177	196/1872	0.010941	0.059945	0.044779	APOD/	6
BP	GO:0010566	regulation of	2/177	17/18723	0.01101	0.060089	0.044887	EGR1/D	2
BP	GO:0010715	regulation of	2/177	17/18723	0.01101	0.060089	0.044887	CST3/L	2
BP	GO:0042326	negative regu	9/177	385/1872	0.011165	0.06082	0.045433	SFRP2/	9

BP	GO:0097553	calcium ion t	5/177	142/1872:	0.011311	0.061494	0.045936	THY1/C	5
BP	GO:0070555	response to i	5/177	143/1872:	0.011633	0.063126	0.047156	CCL19/	5
BP	GO:0033273	response to \	4/177	93/18723	0.011743	0.063325	0.047304	COL1A	4
BP	GO:0002448	mast cell med	3/177	50/18723	0.011759	0.063325	0.047304	PTGDS.	3
BP	GO:0010665	regulation of	3/177	50/18723	0.011759	0.063325	0.047304	SFRP2/	3
BP	GO:0060688	regulation of	3/177	50/18723	0.011759	0.063325	0.047304	SULF1/	3
BP	GO:0014065	phosphatidyl	5/177	144/1872:	0.011962	0.064296	0.048029	DCN/FI	5
BP	GO:0002544	chronic inflar	2/177	18/18723	0.01231	0.065813	0.049162	THBS1/	2
BP	GO:0097709	connective tis	2/177	18/18723	0.01231	0.065813	0.049162	TIMP1/	2
BP	GO:0055074	calcium ion h	10/177	460/1872:	0.012314	0.065813	0.049162	THY1/C	10
BP	GO:0009636	response to t	7/177	262/1872:	0.01246	0.066468	0.049652	SOD3/I	7
BP	GO:0008585	female gonac	4/177	95/18723	0.012621	0.066669	0.049802	NR2F2/	4
BP	GO:0030516	regulation of	4/177	95/18723	0.012621	0.066669	0.049802	FN1/CX	4
BP	GO:0045666	positive regu	4/177	95/18723	0.012621	0.066669	0.049802	CXCL1:	4
BP	GO:0050764	regulation of	4/177	95/18723	0.012621	0.066669	0.049802	MFGE8	4
BP	GO:0010675	regulation of	5/177	146/1872:	0.012638	0.066669	0.049802	IGFBP4	5
BP	GO:0042542	response to h	5/177	146/1872:	0.012638	0.066669	0.049802	COL1A	5
CC	GO:0062023	collagen-cor	64/180	425/1955(	1.57E-60	3.68E-58	2.81E-58	TIMP1/	64
CC	GO:0005788	endoplasmic	32/180	313/1955(	2.56E-24	3.00E-22	2.29E-22	TIMP1/	32
CC	GO:0005581	collagen trim	18/180	87/19550	1.06E-19	8.23E-18	6.30E-18	COL1A	18
CC	GO:0005604	basement me	17/180	96/19550	1.89E-17	1.11E-15	8.46E-16	TIMP1/	17
CC	GO:0098644	complex of c	8/180	21/19550	8.12E-12	3.80E-10	2.90E-10	COL1A	8
CC	GO:0005583	fibrillar collag	6/180	12/19550	4.95E-10	1.57E-08	1.20E-08	COL1A	6
CC	GO:0098643	banded colla	6/180	12/19550	4.95E-10	1.57E-08	1.20E-08	COL1A	6
CC	GO:0031093	platelet alpha	10/180	67/19550	5.37E-10	1.57E-08	1.20E-08	TIMP1/	10
CC	GO:0031091	platelet alpha	11/180	91/19550	7.41E-10	1.93E-08	1.47E-08	TIMP1/	11
CC	GO:0034774	secretory gra	18/180	322/1955(	1.14E-09	2.66E-08	2.03E-08	TIMP1/	18
CC	GO:0060205	cytoplasmic \	18/180	325/1955(	1.32E-09	2.80E-08	2.14E-08	TIMP1/	18
CC	GO:0031983	vesicle lumer	18/180	327/1955(	1.45E-09	2.83E-08	2.16E-08	TIMP1/	18
CC	GO:0043292	contractile fik	12/180	239/1955(	2.30E-06	4.14E-05	3.16E-05	MYL9/(	12
CC	GO:0001527	microfibril	4/180	12/19550	3.25E-06	5.43E-05	4.15E-05	MFAP4	4
CC	GO:0031089	platelet dens	4/180	14/19550	6.47E-06	0.000101	7.72E-05	LGALS:	4
CC	GO:0072562	blood microp	9/180	146/1955(	8.52E-06	0.000124	9.47E-05	C1S/SE	9
CC	GO:0032432	actin filamen	7/180	80/19550	9.00E-06	0.000124	9.47E-05	MYL9/T	7
CC	GO:0030016	myofibril	11/180	231/1955(	1.01E-05	0.000132	0.000101	MYL9/(	11
CC	GO:0005925	focal adhesic	14/180	418/1955(	3.44E-05	0.000423	0.000324	THY1/F	14
CC	GO:0042827	platelet dens	4/180	21/19550	3.68E-05	0.000431	0.000329	LGALS:	4
CC	GO:0030055	cell-substrate	14/180	425/1955(	4.12E-05	0.000451	0.000345	THY1/F	14
CC	GO:0042383	sarcolemma	8/180	138/1955(	4.24E-05	0.000451	0.000345	BGN/C	8
CC	GO:0001725	stress fiber	6/180	71/19550	4.89E-05	0.000477	0.000364	MYL9/T	6
CC	GO:0097517	contractile ac	6/180	71/19550	4.89E-05	0.000477	0.000364	MYL9/T	6
CC	GO:0042641	actomyosin	6/180	81/19550	0.000103	0.000961	0.000735	MYL9/T	6
CC	GO:0030017	sarcomere	9/180	211/1955(	0.000152	0.00137	0.001048	MYL9/T	9
CC	GO:0043202	lysosomal lur	6/180	97/19550	0.000277	0.0024	0.001835	BGN/D	6
CC	GO:0005796	Golgi lumen	6/180	104/1955(	0.000403	0.00337	0.002577	BGN/D	6
CC	GO:0045121	membrane ra	10/180	335/1955(	0.001117	0.008716	0.006665	THY1/F	10
CC	GO:0098857	membrane m	10/180	335/1955(	0.001117	0.008716	0.006665	THY1/F	10
CC	GO:0005775	vacuolar lum	7/180	174/1955(	0.001166	0.0088	0.00673	BGN/D	7
CC	GO:0030018	Z disc	6/180	130/1955(	0.001302	0.009523	0.007283	MYL9/F	6
CC	GO:0031674	I band	6/180	141/1955(	0.00197	0.013968	0.010682	MYL9/F	6
CC	GO:0002102	podosome	3/180	29/19550	0.002352	0.016186	0.012378	GSN/P/	3
CC	GO:0005614	interstitial ma	2/180	10/19550	0.003614	0.02349	0.017963	CCDC8	2
CC	GO:0032593	insulin-respo	2/180	10/19550	0.003614	0.02349	0.017963	LRP1/R	2
CC	GO:0005884	actin filamen	5/180	114/1955(	0.004095	0.025899	0.019806	TPM2/T	5
CC	GO:0009897	external side	10/180	421/1955(	0.00576	0.034851	0.026652	CTSK/T	10
CC	GO:1904813	ficolin-1-rich	5/180	124/1955(	0.005837	0.034851	0.026652	GSN/C:	5
CC	GO:0030426	growth cone	6/180	177/1955(	0.006016	0.034851	0.026652	THY1/F	6
CC	GO:0030864	cortical actin	4/180	79/19550	0.006106	0.034851	0.026652	CALD1.	4

CC	GO:0030427	site of polariz	6/180	183/1955(	0.007046	0.039256	0.03002	THY1/F	6
CC	GO:0005901	caveola	4/180	84/19550	0.007569	0.041191	0.0315	TFPI/C/	4
MF	GO:0005201	extracellular	41/175	172/1836{	2.58E-46	7.68E-44	6.37E-44	COL1A	41
MF	GO:0005539	glycosamino	24/175	230/1836{	2.63E-18	3.91E-16	3.24E-16	BGN/D	24
MF	GO:0030020	extracellular	13/175	41/18368	4.74E-17	4.70E-15	3.89E-15	COL1A	13
MF	GO:0005518	collagen binc	15/175	69/18368	9.55E-17	7.09E-15	5.88E-15	DCN/C	15
MF	GO:0008201	heparin bind	20/175	166/1836{	1.20E-16	7.12E-15	5.91E-15	SOD3/I	20
MF	GO:1901681	sulfur compc	21/175	265/1836{	9.80E-14	4.85E-12	4.02E-12	SOD3/I	21
MF	GO:0005178	integrin bind	16/175	144/1836{	5.51E-13	2.34E-11	1.94E-11	COL3A	16
MF	GO:0061134	peptidase re	19/175	230/1836{	7.49E-13	2.78E-11	2.31E-11	TIMP1/	19
MF	GO:0048407	platelet-deri	7/175	11/18368	2.02E-12	6.66E-11	5.53E-11	COL1A	7
MF	GO:0019838	growth facto	15/175	141/1836{	5.70E-12	1.69E-10	1.40E-10	COL1A	15
MF	GO:0050840	extracellular	10/175	56/18368	1.16E-10	3.13E-09	2.60E-09	BGN/D	10
MF	GO:0061135	endopeptida	15/175	194/1836{	5.49E-10	1.36E-08	1.13E-08	TIMP1/	15
MF	GO:0004866	endopeptida	14/175	180/1836{	1.95E-09	4.46E-08	3.70E-08	TIMP1/	14
MF	GO:0030414	peptidase inł	14/175	187/1836{	3.21E-09	6.81E-08	5.65E-08	TIMP1/	14
MF	GO:0001968	fibronectin b	7/175	28/18368	6.32E-09	1.19E-07	9.88E-08	CTSK/S	7
MF	GO:0002020	protease binc	12/175	135/1836{	6.41E-09	1.19E-07	9.88E-08	TIMP1/	12
MF	GO:0008191	metalloendo	5/175	16/18368	2.97E-07	5.20E-06	4.31E-06	TIMP1/	5
MF	GO:0043394	proteoglycan	6/175	36/18368	1.05E-06	1.74E-05	1.44E-05	FN1/CT	6
MF	GO:0008009	chemokine a	6/175	49/18368	6.83E-06	0.000107	8.86E-05	CXCL9/	6
MF	GO:0005520	insulin-like g	5/175	29/18368	7.31E-06	0.000109	9.01E-05	IGFBP7	5
MF	GO:0003779	actin binding	15/175	441/1836{	2.20E-05	0.000311	0.000258	CALD1	15
MF	GO:0030021	extracellular	4/175	22/18368	5.09E-05	0.000687	0.00057	BGN/D	4
MF	GO:0016504	peptidase ac	5/175	43/18368	5.32E-05	0.000687	0.00057	FN1/SF	5
MF	GO:0042379	chemokine re	6/175	72/18368	6.37E-05	0.000789	0.000654	CXCL9/	6
MF	GO:0048018	receptor liga	15/175	487/1836{	6.82E-05	0.00081	0.000671	TIMP1/	15
MF	GO:0030546	signaling rec	15/175	495/1836{	8.18E-05	0.000934	0.000775	TIMP1/	15
MF	GO:0097493	structural mc	3/175	11/18368	0.000133	0.001459	0.00121	FBN1/E	3
MF	GO:0008083	growth facto	8/175	162/1836{	0.000164	0.001744	0.001446	TIMP1/	8
MF	GO:0031994	insulin-like g	3/175	13/18368	0.000227	0.002321	0.001925	IGFBP4	3
MF	GO:0004252	serine-type e	8/175	174/1836{	0.000268	0.00265	0.002198	C1S/CT	8
MF	GO:0004867	serine-type e	6/175	98/18368	0.00035	0.003355	0.002782	SERPIN	6
MF	GO:0008236	serine-type p	8/175	191/1836{	0.0005	0.004638	0.003846	C1S/CT	8
MF	GO:0017171	serine hydrol	8/175	195/1836{	0.000573	0.005157	0.004277	C1S/CT	8
MF	GO:0045236	CXCR chemo	3/175	18/18368	0.000624	0.005454	0.004523	CXCL9/	3
MF	GO:0008307	structural cor	4/175	42/18368	0.000672	0.005701	0.004728	MYL9/T	4
MF	GO:0046332	SMAD bindir	5/175	79/18368	0.000946	0.007803	0.006471	COL1A	5
MF	GO:0043236	laminin bindi	3/175	28/18368	0.002337	0.018762	0.01556	LGALS1	3
MF	GO:0098631	cell adhesion	4/175	59/18368	0.00241	0.018832	0.015618	PALLD/	4
MF	GO:0017147	Wnt-protein	3/175	30/18368	0.002857	0.021755	0.018042	SFRP2/	3
MF	GO:0005516	calmodulin b	7/175	201/1836{	0.003188	0.023672	0.019633	CALD1	7
MF	GO:0005126	cytokine rece	8/175	271/1836{	0.004529	0.032804	0.027206	CXCL9/	8
MF	GO:0042805	actinin bindir	3/175	36/18368	0.004818	0.03345	0.027742	PALLD/	3
MF	GO:0051015	actin filamen	7/175	217/1836{	0.004843	0.03345	0.027742	TAGLN	7
MF	GO:0005125	cytokine acti	7/175	235/1836{	0.007401	0.049117	0.040735	TIMP1/	7
MF	GO:0001664	G protein-co	8/175	295/1836{	0.007442	0.049117	0.040735	CXCL9/	8
MF	GO:0071837	HMG box do	2/175	14/18368	0.007618	0.049185	0.040791	PRRX1/	2
MF	GO:0004175	endopeptida	10/175	430/1836{	0.008326	0.052614	0.043635	C1S/CT	10
MF	GO:0051371	muscle alpha	2/175	15/18368	0.008735	0.054049	0.044825	PALLD/	2
MF	GO:0016641	oxidoreducta	2/175	16/18368	0.009921	0.060133	0.049871	VCAM1	2
