# Supplementary material for: Development of a Cancer-Associated Fibroblast-Related Prognostic Model in Breast Cancer via Bulk and Single-Cell RNA Sequencing
Source: Biomed Res Int. 2022 Dec 2;2022:2955359. doi: 10.1155/2022/2955359 (PMC9735320; doi:10.1155/2022/2955359)
Supplement: Supplementary 3 — Supplementary Table S2: KEGG enrichment findings. [file 2955359.f3.pdf]

ID	Description	GeneRat	BgRatio	pvalue	p.adjust	qvalue	geneID	Count
hsa04510	Focal adhesio	20/91	201/8112	4.14E-14	7.075E-12	5.9231E-12	COL1A1/CC	20
hsa04974	Protein digest	13/91	103/8112	8.71E-11	7.4442E-09	6.2321E-09	COL1A1/CC	13
hsa04512	ECM-receptor	12/91	88/8112	1.94E-10	1.1031E-08	9.2346E-09	COL1A1/CC	12
hsa04933	AGE-RAGE sig	11/91	100/8112	1.2E-08	5.1128E-07	4.2803E-07	COL1A1/CC	11
hsa04151	PI3K-Akt signa	17/91	354/8112	3.08E-07	1.0523E-05	8.8094E-06	COL1A1/CC	17
hsa04610	Complement a	9/91	85/8112	3.86E-07	1.0991E-05	9.2012E-06	C1S/SERPIN	9
hsa04926	Relaxin signali	9/91	129/8112	1.28E-05	0.00031335	0.00026233	COL1A1/CC	9
hsa05165	Human papillo	14/91	331/8112	1.66E-05	0.00032357	0.00027088	COL1A1/CC	14
hsa05146	Amoebiasis	8/91	102/8112	1.7E-05	0.00032357	0.00027088	COL1A1/CC	8
hsa05205	Proteoglycans	10/91	205/8112	9.22E-05	0.00157581	0.00131923	COL1A1/DC	10
hsa04810	Regulation of	10/91	218/8112	0.000153	0.0023835	0.00199542	MYL9/FN1/	10
hsa05133	Pertussis	6/91	76/8112	0.000196	0.00279961	0.00234378	C1S/SERPIN	6
hsa05144	Malaria	5/91	50/8112	0.000226	0.002977	0.00249228	CCL2/THBS	5
hsa04061	Viral protein i	6/91	100/8112	0.000864	0.01055382	0.00883546	CXCL9/CXC	6
hsa04670	Leukocyte tra	6/91	114/8112	0.001706	0.01944625	0.01628003	MYL9/THY1	6
hsa05410	Hypertrophic	5/91	90/8112	0.003304	0.03531089	0.02956159	TPM2/TPM	5
hsa05323	Rheumatoid a	5/91	93/8112	0.003806	0.0364932	0.0305514	CTSK/CCL2/	5
hsa04270	Vascular smo	6/91	134/8112	0.003841	0.0364932	0.0305514	MYL9/CALD	6
hsa05150	Staphylococcu	5/91	96/8112	0.00436	0.03700203	0.03097739	C1S/C1R/C	5
hsa05414	Dilated cardio	5/91	96/8112	0.00436	0.03700203	0.03097739	TPM2/TPM	5
hsa05418	Fluid shear str	6/91	139/8112	0.004596	0.03700203	0.03097739	MMP2/CCL	6
hsa01522	Endocrine res	5/91	98/8112	0.00476	0.03700203	0.03097739	MMP2/FOS	5
hsa04620	Toll-like recep	5/91	104/8112	0.006118	0.04548306	0.03807754	CTSK/CXCL	5
