# Supplementary material for: Development of a Cancer-Associated Fibroblast-Related Prognostic Model in Breast Cancer via Bulk and Single-Cell RNA Sequencing
Source: Biomed Res Int. 2022 Dec 2;2022:2955359. doi: 10.1155/2022/2955359 (PMC9735320; doi:10.1155/2022/2955359)
Supplement: Supplementary 4 — Supplementary Table S3: list of 193 cancer-associated fibroblasts genes. [file 2955359.f4.pdf]

fibroblasts.markergenes
TIMP1
COL1A1
BGN
DCN
COL1A2
MYL9
COL3A1
CALD1
C1S
COL6A2
TAGLN
SOD3
LUM
APOD
IGFBP7
IGFBP4
SPARC
RGS5
ACTA2
SERPINF1
SERPING1
COL6A1
FN1
CTSK
SFRP2
THY1
CCDC80
POSTN
CXCL9
NDUFA4L2
MFGE8
COL6A3
IFITM3
ISLR
PRSS23
CXCL14
PCOLCE
RGS16
LGALS3BP
CCL19
RARRES2
FRZB
CTGF
AEBP1
CD248
SPARCL1
NNMT
IGFBP5
TIMP3

GEM
PDGFRB
GSN
C1R
EGR1
CTHRC1
SELM
CPE
CYR61
TPM2
VCAN
LHFP
PTGDS
C3
MMP11
TPM1
COL5A2
PALLD
LGALS1
MMP2
CST3
FILIP1L
CCL2
COL18A1
CRISPLD2
CYP1B1
CXCL12
PRRX1
GGT5
MXRA8
FBLN1
CFD
COL4A2
PTRF
PLPP3
PRKCDBP
C11orf96
OLFML3
FSTL1
SPON2
SERPINH1
CNN3
MFAP4
MYLK
FOS
EFEMP2
NOTCH3
MXRA5
TGFB1I1
SULF1

IGFBP6
VIM
PMP22
PLAC9
RASD1
IFI27
RARRES1
SMOC2
TFPI
ADAMTS4
MAP1B
FBN1
IFIT3
MDK
COL4A1
DKK3
C10orf10
TMEM176B
ADIRF
DPT
PLXDC1
TMEM176A
C1QTNF1
GBP1
PPIC
THBS2
HTRA3
NR2F2
SLIT3
CDH11
SEPT4
UACA
EDNRA
JUN
COX4I2
ACTN1
SEPP1
LMNA
TIMP2
EFEMP1
CAV1
LRP1
IFI6
EMILIN1
CLEC11A
PGF
CFH
MFAP2
COL12A1
NUPR1

TCF4
CSRP1
THBS1
EPS8
CEBPD
CERCAM
DSTN
COL14A1
SYNPO2
ATF3
BST2
MIR4435-2HG
COL5A1
F2R
SEPT11
FBLN2
ID3
SSPN
FGF7
GJA4
ADAMTS1
EHD2
A2M
S100A13
VCAM1
CILP
MCAM
CRYAB
GUCY1A3
LAMA4
CST1
MT2A
LOXL1
KCNJ8
MYO1B
MGP
ITGA1
PPP1R14A
EPAS1
ECM1
CXCL11
TSC22D1
IGF1
RAB13
